# Unravelling Site-Specific Photo-Reactions of Ethanol on Rutile TiO_2_(110)

**DOI:** 10.1038/srep21990

**Published:** 2016-02-26

**Authors:** Jonas Ø. Hansen, Regine Bebensee, Umberto Martinez, Soeren Porsgaard, Estephania Lira, Yinying Wei, Lutz Lammich, Zheshen Li, Hicham Idriss, Flemming Besenbacher, Bjørk Hammer, Stefan Wendt

**Affiliations:** 1 Interdisciplinary Nanoscience Center (iNANO), Department of Physics and Astronomy, and Institute for Storage Ring Facilities (ISA), Aarhus University, DK-8000 Aarhus C, Denmark; 2 SABIC-Centre for Research and Development (CRD) at KAUST, P.O. Box 4545-4700, Thuwal 23955, Saudi Arabia

## Abstract

Finding the active sites of catalysts and photo-catalysts is crucial for an improved fundamental understanding and the development of efficient catalytic systems. Here we have studied the photo-activated dehydrogenation of ethanol on reduced and oxidized rutile TiO_2_(110) in ultrahigh vacuum conditions. Utilizing scanning tunnelling microscopy, various spectroscopic techniques and theoretical calculations we found that the photo-reaction proceeds most efficiently when the reactants are adsorbed on regular Ti surface sites, whereas species that are strongly adsorbed at surface defects such as O vacancies and step edges show little reaction under reducing conditions. We propose that regular Ti surface sites are the most active sites in photo-reactions on TiO_2_.

Catalysis and photo-catalysis are fields of paramount importance both with a view on the demands on the chemical industry and the challenges in future renewable energy generation as well as to sustain our environment. To fulfil all these demands it is crucial to enhance the efficiency of designated chemical processes. One of the most promising materials in photo-catalysis is Titania (TiO_2_)^1^,^2^,^3^,^4^,^5^,^6^. Besides photo-catalysis, TiO_2_ is used in thermal heterogeneous catalysis, solar cells, gas sensors, and biocompatible materials.

An increasingly important field is the photo-catalytic and renewable generation of fuels, and particularly ethanol (CH_3_CH_2_OH or EtOH) holds great promise in this regard^5^,^6^. In addition, EtOH is an essential solvent and it could be used as feedstock in a possible green chemistry in the future^7^,^8^,^9^. These expectations, and the fact that alcohols serve as model molecules of catalytic and photo-catalytic processes^4^,^10^,^11^,^12^, have stimulated considerable research efforts towards the thermal and photo-catalytic oxidation of EtOH on TiO_2_. Numerous studies have been conducted using TiO_2_ powders, where TiO_2_ nanoparticles (often a mix of the two most common polymorphs, anatase and rutile) were used to oxidize EtOH condensed as liquid or in the gas phase^13^,^14^,^15^,^16^,^17^,^18^,^19^,^20^,^21^. While other work has addressed photo-catalytic reactions of methanol (MeOH)^4^,^6^,^11^,^22^,^23^,^24^,^25^,^26^ it is worth indicating two fundamental differences between MeOH and EtOH. First, because of the carbon-carbon bond the chemistry of EtOH is akin to higher hydrocarbons and different from that of C1 hydrocarbons. Second, MeOH is made from syngas and therefore not a renewable feedstock.

In surface science, where single crystalline surfaces can be studied at ultrahigh vacuum (UHV) conditions, particular focus has been on the interaction between EtOH and the rutile TiO_2_(110)–(1 × 1) surface^27^,^28^,^29^,^30^,^31^,^32^,^33^,^34^,^35^,^36^,^37^,^38^,^39^, because this surface is the most stable one of rutile and often serves as a model for transition metal surfaces^1^,^2^,^3^,^4^,^10^,^11^,^12^. The TiO_2_(110)–(1 × 1) surface consists of alternating rows of fivefold-coordinated titanium (5f-Ti) atoms (the Ti troughs) and protruding, twofold coordinated bridge-bonded oxygen (O_br_) atoms. Following cycles of Ar^+^ sputtering and vacuum-annealing, the TiO_2_(110) crystals are reduced, leading to the creation of bulk defects and O_br_ vacancies on the surface^1^,^10^,^12^,^40^,^41^. This leads to changes in electronic properties, where the empty Ti*3d* orbitals become populated, leading to a state within the ~3.1 eV wide band gap ~0.85 eV below the Fermi level (E_F_)^1^,^40^,^41^,^42^. This Ti*3d* defect state can be removed upon oxygen adsorption^1^,^10^,^40^,^41^,^42^,^43^.

The adsorption of EtOH and the thermally activated chemistry of EtOH on rutile TiO_2_(110)–(1 × 1) have been studied previously by means of photoelectron spectroscopy (PES)^28^,^30^,^44^, temperature-programmed desorption (TPD)^27^,^28^,^29^,^32^,^33^,^36^,^37^,^38^,^39^, femtosecond two-photon photoemission spectroscopy (2PPE)^36^, and scanning tunnelling microscopy (STM)^32^,^33^,^35^. In addition, the EtOH–TiO_2_(110) interaction has been studied using density functional theory (DFT) calculations^31^,^32^,^33^,^34^,^35^,^36^. The adsorption of an EtOH molecule occurs in a manner similar to that of acid–base reaction whereby the oxygen of EtOH is adsorbed on a 5f-Ti site. Ambiguity still exists to which extents EtOH adsorbs molecularly and dissociatively^32^,^36^.

Some studies also addressed the photo-chemistry of EtOH on rutile TiO_2_(110)^30^,^31^,^36^,^37^,^38^,^39^. PES and TPD studies suggest that EtOH converts to acetaldehyde (CH_3_CHO) and, finally, acetate (CH_3_COO^−^) under UV light illumination in an oxygen background^30^,^31^. A 2PPE study also revealed the formation of CH_3_CHO and reports on a photo-induced excited state ~2.4 eV above E_F_ for EtOH/TiO_2_(110) that was associated with the dissociation of EtOH on 5f-Ti sites^36^. Finally, applying pump–probe laser ionization techniques, it has been suggested that, in the presence of oxygen, methyl radicals are produced during the photo-catalytic oxidation of EtOH^37^.

Here we studied the photo-activated dehydrogenation of EtOH on TiO_2_(110) in different oxidation states by combining several surface science techniques and DFT calculations. The active sites on the terraces and at step edges are identified by high-resolution STM, whereas the chemical identity of the products is unravelled by TPD and isothermal mass spectrometry (ISOMS) measurements. Utilizing synchrotron-radiation PES, we compare the rates of EtOH dehydrogenation at 290 K for TiO_2_(110) surfaces in different oxidation states. A reaction mechanism is tested with the help of DFT calculations that explains the experimental observations.

## Results

### Photo-Reactions of EtOH on Rutile TiO_2_(110) studied by STM

**Figure 1:**
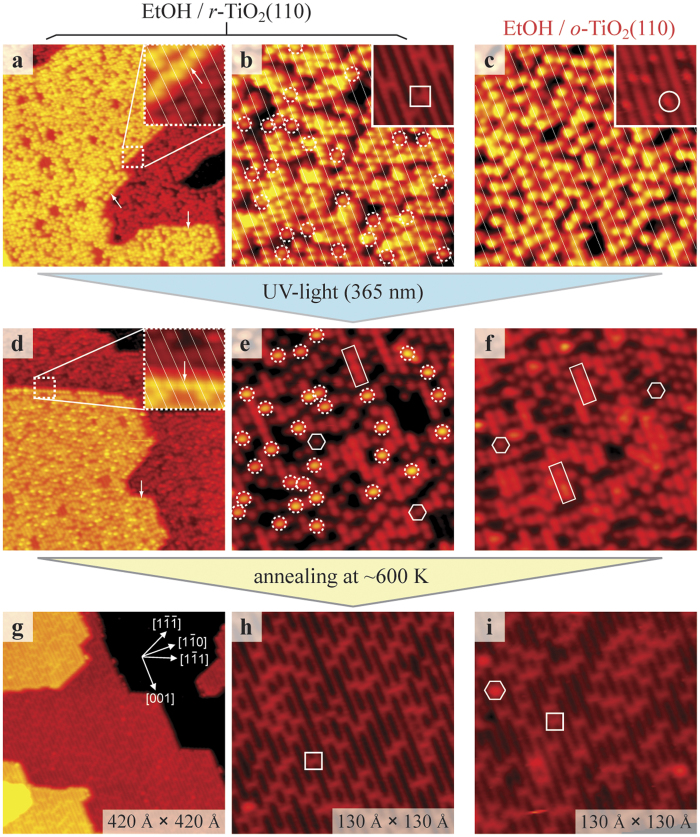
STM study of EtOH photo-reactions on *r*- and *o*-TiO_2_(110) surfaces. (**a**) STM image (420 Å × 420 Å) obtained after exposing an *r*-TiO_2_(110) surface to EtOH at 300 K. (**b**) Zoom-in STM image (130 Å × 130 Å) corresponding to the data in (a) that allows to distinguish between EtOH_Ti_/EtO_Ti_ and EtO_br_ species. (**c**) Zoom-in STM image (130 Å × 130 Å) obtained after exposing an *o*-TiO_2_(110) surface to EtOH at 300 K. Insets in (**b**,**c**) display (40 Å × 40 Å) areas on the pristine *r*- and *o*-TiO_2_(110) surfaces. (**d**–**f**) Corresponding STM images obtained after illumination of the EtOH-covered surfaces with UV-light for 11 min at 290 K in UHV. Insets in (**a,d**) show the indicated areas (40 Å × 40 Å) enlarged. (**g**–**i**) Corresponding STM images obtained after subsequent annealing of the TiO_2_(110) crystals for 2 min at ~550 K (**g,h**) and ~630 K (**i**), respectively. Symbols indicate O_br_ vacancies (squares), an O_ot_ adatom (circle), EtO_br_ ethoxides (dotted circles), EtO_S_ ethoxides (arrows), isolated H_ad_ species (hexagons), and rows of H_ad_ species (rectangles), respectively. The Ti troughs are indicated in (**b,c**) and in the insets of (**a,d**) by thin white lines. STM images were collected with a tunnelling current ≤0.1 nA and a tunnelling voltage of ~1.2 V. The STM images are shown enlarged in the Supplementary Information (Figs S1–S9).

To test the influence of the oxidation state and the surface structure on the photo-reactivity of EtOH we prepared TiO_2_(110) samples with different characteristic point defects on the surface. Among the reduced TiO_2_(110) surfaces we distinguish between clean, reduced TiO_2_(110) surfaces [*r*-TiO_2_] characterized by the presence of O_br_ vacancies and hydrogenated TiO_2_(110) surfaces [*h*-TiO_2_] characterized by H adatoms (H_ad_), capping some of the O_br_ atoms. Starting with a clean *r*-TiO_2_(110) surface, an *h*-TiO_2_(110) surface can be easily produced by letting water dissociate at the O_br_ vacancies^43^,^45^,^46^,^47^,^48^. Additionally, we prepared oxidized TiO_2_(110) surfaces [*o*-TiO_2_] by exposing *h*-TiO_2_(110) surfaces to 200 L O_2_ [1 L (Langmuir) = 1.33 × 10^−6^ mbar · s] at 300 K^10^,^41^. Such *o*-TiO_2_(110) surfaces are characterized by a diminished Ti*3d* gap state, by perfect O_br_ rows (no O_br_ vacancies) and by a number of O adatoms (O_ot_) located on-top of 5f-Ti sites in the Ti troughs (Fig. 1c, inset). The O_ot_ adatoms show up in the STM images as protrusions distributed along the Ti troughs^10^,^41^,^46^,^49^,^50^. In empty-state STM images of TiO_2_(110), the Ti troughs appear bright, whereas geometrically protruding O_br_ atoms appear dark^1^,^10^,^46^. Accordingly, the O_br_ vacancies on *r*-TiO_2_(110) appear as protrusions along the dark rows, “connecting” bright Ti troughs (Fig. 1b, inset).

Following the saturation of an *r*-TiO_2_(110) surface with EtOH at 300 K [Fig. 1a,b: EtOH/*r*-TiO_2_(110)] we found bright protrusions on (34 ± 2)% of the 5f-Ti sites that arise both from EtOH_Ti_ molecules and EtO_Ti_ ethoxides (EtOH_Ti_/EtO_Ti_)^32^. The EtOH_Ti_ molecules and EtO_Ti_ ethoxides appear in the STM images with very similar contrast^32^, allowing their distinction solely at low coverage. In addition, adsorbates occurred in the O_br_ rows (marked by dotted circles in Fig. 1b) that are ascribed to EtO_br_ ethoxides, resulting from the dissociation of EtOH_Ti_ molecules at O_br_ vacancies^32^. New adsorbates also occurred along the step edges running parallel to 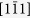 and 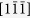 directions (Fig. 1a, inset), which are ascribed as EtO_S_ ethoxides (“S” stands for step)^33^. The EtO_S_ ethoxides are adsorbed at the upper terraces and located at the end of the O_br_ rows^33^. Both ethoxide species (EtO_br_ and EtO_S_) are strongly bound at O vacancies^32^,^33^.

When starting instead with an *o*-TiO_2_(110) surface, we again obtained EtOH_Ti_ molecules and EtO_Ti_ ethoxides in the Ti troughs [Fig. 1c: EtOH/*o*-TiO_2_(110)]. However, this time no EtO_br_ ethoxides were formed, because no O_br_ vacancies existed at which the EtOH molecules could dissociate. In addition, on EtOH/*o*-TiO_2_(110), the density of EtO_S_ ethoxides was considerably lower than on EtOH/*r*-TiO_2_(110). On EtOH/*o*-TiO_2_(110), the density of O_ot_ adatoms was (10.1 ± 0.5)%ML [1 ML (monolayer) is the density of the (1 × 1) unit cells, 5.2 × 10^14^/cm^2^] and the coverage of EtOH_Ti_/EtO_Ti_ species was (24.1 ± 0.8)% ML. Because the EtOH_Ti_ and EtO_Ti_ species protrude much more from the surface than the O_ot_ adatoms^32^,^50^ the O_ot_ adatoms cannot be seen in the STM images. However, before the sample was exposed to EtOH, the O_ot_ adatoms were well-resolved in the STM images (Fig. 1c, inset). Thus, we analysed the images acquired on *o*-TiO_2_(110) before the EtOH exposure to estimate the O_ot_ adatom density.

Following UV-light illumination for 11 min at 290 K [365 nm; ~2 × 10^16^ photons/(s · cm^2^)] we obtained clear changes on both EtOH-covered TiO_2_(110) surfaces, as illustrated by the STM images shown in Fig. 1d–f. Importantly, all the adsorbates in the Ti troughs, EtOH_Ti_ and EtO_Ti_ species, disappeared on EtOH/*r*-TiO_2_ (Fig. 1d,e) and EtOH/*o*-TiO_2_ (Fig. 1f). Solely the EtO_br_ and EtO_S_ ethoxides on *r*-TiO_2_(110) did not (or rarely) photo-react (in Fig. 1b,e all the EtO_br_ species are marked by white dotted circles). In addition, H_ad_ species were evident on both surfaces in very high densities [*r*-TiO_2_: (27 ± 1)%ML; *o*-TiO_2_: (35 ± 1)%ML]. Single H_ad_ species appear brighter than O_br_ vacancies^10^,^12^,^46^,^47^ (some are marked in Fig. 1e,f,i by white hexagons). Note that H_ad_ species adsorbed closely to each other within the O_br_ rows appear as extra-bright, elongated protrusions (see the examples indicated by the rectangles in Fig. 1e,f). Regarding the EtOH/*o*-TiO_2_(110) surface, notice that no O_ot_ adatoms remained on the surface after illumination with UV light (Fig. 1f). This can be explained through the recombination of H_ad_ species (created in the course of the photo-reaction) with O_ot_ adatoms^10^,^43^,^51^. Thus, water molecules are formed in the photo-reaction. These water molecules greatly facilitate the diffusion of H_ad_ species^47^,^48^, which explains why all the O_ot_ adatoms were reacted off. Because we observed only few water monomers^52^ in the STM images in Fig. 1d–f, many of them must have desorbed from the surface: 2H_ad_+O_ot_ → H_2_O ↑.

Subsequent annealing of the two TiO_2_(110) samples at 550–630 K for 2 min led to *r*-TiO_2_(110) surfaces without any H_ad_ species, regardless whether we started the experiment with *r*- or *o*-TiO_2_(110), see Fig. 1g–i. Interestingly, the *r*-TiO_2_(110) surfaces obtained after brief annealing at this high temperature were characterized by O_br_ vacancy densities that were about twice as high as those found on the clean *r*-TiO_2_(110) surfaces at the very beginning of the experiments. These enhanced O_br_ vacancy densities originate from the recombinative desorption of H_ad_ species – a reaction that includes the formation of water molecules and the consumption of O_br_ atoms: 2H_ad_+O_br_ → H_2_O ↑^43^,^45^. The formation of H_ad_ species within the photo-reaction of EtOH_Ti_ and EtO_Ti_ species is confirmed by these annealing-experiments. Additional STM studies (Fig. S10 in the Supplementary Information) and water-TPD measurements (Fig. S11) further corroborate the formation of H_ad_ species in the photo-reaction on EtOH/*r*-TiO_2_(110).

### Evidence for acetaldehyde formation by mass spectroscopy

**Figure 2:**
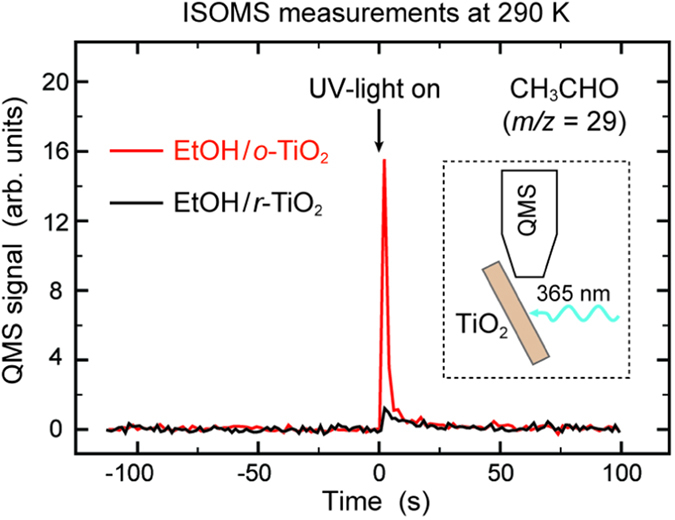
ISOMS data acquired during EtOH photo-reaction at 290 K. Black curve: *r*-TiO_2_(110); red curve: *o*-TiO_2_(110). Prior to the experiments both TiO_2_(110) surfaces were saturated with EtOH at 300 K. The inset displays the experimental setup. The quadrupole mass spectrometer (QMS), the TiO_2_(110) crystal (light brown block) and the direction of the incident light are indicated.

When illuminating EtOH/*r*-TiO_2_(110) and EtOH/*o*-TiO_2_(110) surfaces in UHV, the H_ad_ species are the sole detectable reaction product on the surfaces (Fig. 1a–f). This finding implies that all other products are ejected into the vacuum immediately after their formation. To identify these products we conducted time-resolved ISOMS experiments (Fig. 2). In such experiments, the reaction products are detected with a mass spectrometer *in-situ* as a function of time and at a given sample temperature. The experimental setup is sketched in the inset of Fig. 2. During the ISOMS experiments the sample was held at 290 K (as in the STM experiments) and masses *m*/*z* = 18; 26; 29; 31; 43; 44 were recorded. Regardless whether we started with EtOH-saturated *r*-, *h*- or *o*-TiO_2_(110) surfaces, no *m*/*z* = 31 peak was detected, but we always detected a peak for *m*/*z* = 29 when the light was switched on. Because the base peak of EtOH is *m*/*z* = 31, simple photo-desorption of the EtOH species in the Ti troughs can be ruled out. Instead, on the basis of the known cracking pattern of acetaldehyde^53^, we conclude that the *m*/*z* = 29 signals originate from acetaldehyde (CH_3_CHO). The *m*/*z* = 29 signal obtained for EtOH/*o*-TiO_2_(110) was more intense than that obtained for EtOH-covered *h*- and *r*-TiO_2_(110) surfaces [Fig. 2, data for EtOH/*h*-TiO_2_(110) (not shown) are very similar to those found for EtOH/*r*-TiO_2_(110)]. In each case the *m*/*z* = 29 signals dropped down to the base line in less than 1 min illumination time, indicating that the EtOH photo-reactions have completed.

### EtOH photo-reaction on *r*-TiO_2_(110) in the presence of oxygen

**Figure 3:**
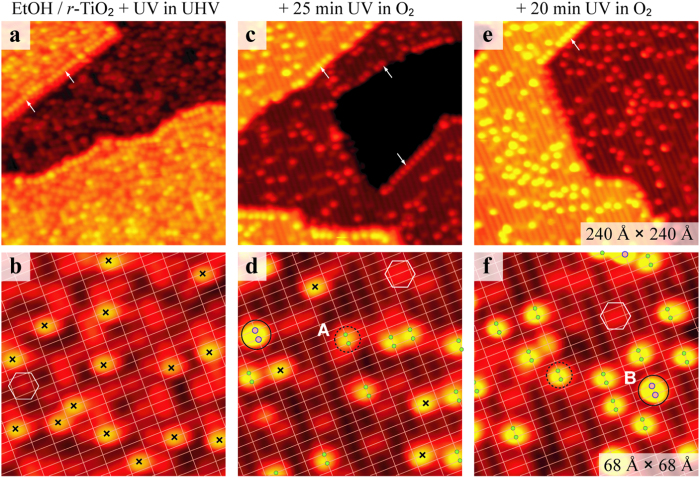
STM study of the photo-reaction on EtOH/*r*-TiO_2_(110) in oxygen (fate of EtO_br_ ethoxides). (**a,b**) STM images (240 Å × 240 Å and 68 Å × 68 Å, respectively) of an illuminated EtOH/*r*-TiO_2_(110) surface. The UV-light illumination was accomplished at 290 K in UHV. (**c,d**) STM images of the same sample after illumination in 5 × 10^−8^ mbar O_2_ at 290 K for 25 min. (**e,f**) STM images of the same sample after additional illumination in 5 × 10^−8^ mbar O_2_ at 290 K for 20 min. Symbols indicate H_ad_ species (hexagons), EtO_S_ ethoxides (white arrows) and EtO_br_ ethoxides (black crosses). Occupied adsorption sites of unidentified products “A” (dotted black circle) and “B” (black circle) are indicated by green and pink dots, respectively. Lattice grids in (**b**,**d,f**) are centred on-top of 5f-Ti sites.

In contrast to EtOH_Ti_ and EtO_Ti_ species, the EtO_br_ and EtO_S_ ethoxides strongly bound to O vacancies on EtOH/*r*-TiO_2_(110) did barely photo-react in UHV (Fig. 1b,e). An analysis of STM images acquired before and after UV-light illumination for 11 min revealed no clear changes in the densities of EtO_br_ and EtO_S_ ethoxides. Nevertheless, a 30 min illumination with UV light decreased the density of EtO_br_ ethoxides by ~34%. To photo-react more of the EtO_br_ and EtO_S_ ethoxide species we conducted another STM experiment wherein the photo-reaction was carried out in an O_2_ background. This experiment was started with a TiO_2_(110) surface with EtO_br_/EtO_S_ ethoxides and H_ad_ species prepared by illuminating an EtOH/*r*-TiO_2_(110) surface with UV-light for 7 min (Fig. 3a,b). The STM images in Fig. 3a,b are alike those presented in Fig. 1d,e because the corresponding sample preparations were almost identical. Subsequently, the sample was illuminated in ~5 × 10^−8^ mbar O_2_ for 25 min at 290 K (Fig. 3c,d) and another 20 min at the same O_2_ pressure and temperature (Fig. 3e,f). In O_2_, the EtO_br_ ethoxide species photo-reacted indeed more efficiently, as was evident from there diminished occurrence. For example, within 25 min the density of EtO_br_ ethoxides decreased from (6.1 ± 0.2) to (2.2 ± 0.1)%ML. At the same time, we found that two new types of adsorbates (denoted “A” and “B”) had appeared in the Ti troughs. In Fig. 3d,f, we indicate some of the newly formed adsorbates in the Ti troughs. Along the [001] direction, these reaction products are centred in between two 5f-Ti sites. After a total illumination time of 45 min only (0.5 ± 0.1)%ML EtO_br_ ethoxides remained. The densities of the new reaction products in the Ti troughs (sum of both types) were (4.0 ± 0.3)%ML and (6.3 ± 0.4)%ML for 25 and 45 min total illumination time, respectively. Clearly, the photo-reactivity of the EtO_br_ species is considerably higher in an O_2_ background than in UHV, and new products are formed in an O_2_ background that are stable on the TiO_2_(110) surface at 290 K.

**Figure 4:**
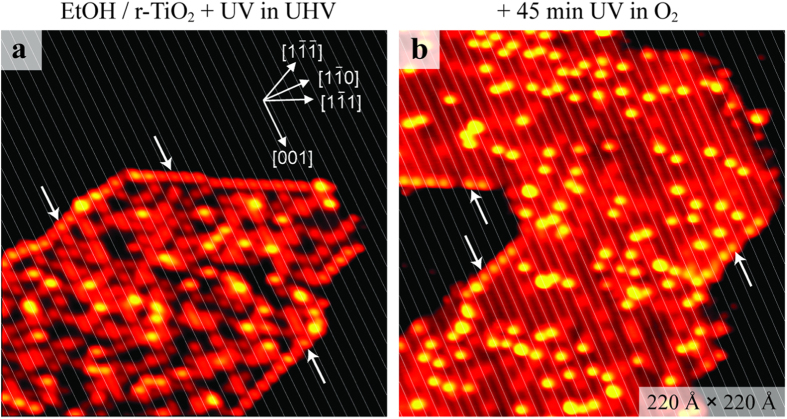
STM study of the photo-reaction on EtOH/*r*-TiO_2_(110) in oxygen (fate of EtO_S_ ethoxides). (**a**) STM image (220 Å × 220 Å) of an illuminated EtOH/*r*-TiO_2_(110) surface. The UV-light illumination was accomplished at 290 K in UHV. Few EtO_S_ ethoxides are indicated by white arrows. (**b**) STM image of the same sample after illumination in 5 × 10^−8^ mbar O_2_ at 290 K for 45 min. White lines are superimposed on the Ti troughs. These STM images are shown with higher contrast than the images in the other figures.

The EtO_S_ ethoxides, however, are even more difficult to photo-react than the EtO_br_ ethoxides (Fig. 4). In the experiment corresponding to Fig. 4a,b, we illuminated an EtOH/*r*-TiO_2_(110) surface with UV light in ~5 × 10^−8^ mbar O_2_ for 45 min. It can be seen that the species adsorbed along the step edges running in 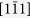 and 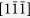 directions (some are marked by white arrows) appear in between the Ti troughs, i.e. at the end of the O_br_ rows on the upper terraces. This observation confirms the assignment of these protrusions to EtO_S_ ethoxides^33^. Notably, the occurrence of the EtO_S_ ethoxides did not decline, in spite of the long illumination time in the presence of oxygen.

Another aspect regarding the EtOH photo-reaction experiments in O_2_ worth noting is the lowered coverage of H_ad_ species. The STM images depicted in Fig. 3 and 4 show this quite clearly. Apparently, oxygen reacts the H_ad_ species off, forming water. Not all of the H_ad_ species reacted at the chosen conditions (see Fig. 3f and 4b).

### EtOH photo-reactions on *o*- and *h*-TiO_2_(110) surfaces studied by PES

**Figure 5:**
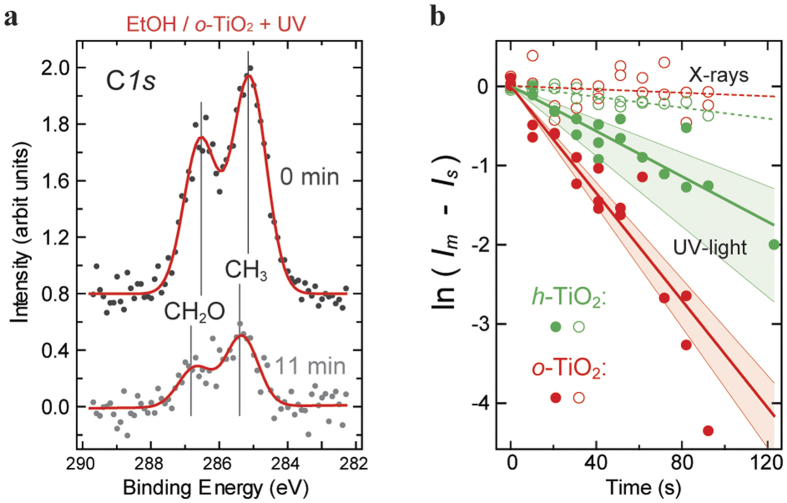
Photo-reactions on EtOH/*o*-TiO_2_(110) and EtOH/*h*-TiO_2_(110) surfaces studied by PES. (**a**) C*1s* PES data (dots) collected with photon energy (*hν*) of 350 eV on EtOH/*o*-TiO_2_(110) following UV light illumination at 290 K for 0 and 11 min, respectively. Spectra are offset for clarity. Gaussian fits to the data are shown as red curves. (**b**) Logarithmic plot of the normalized integrated areas of the C*1s* spectra as function of illumination time (filled green and red dots) and X-rays (open green and red dots), respectively. Starting points in these experiments were *h*- (green) and *o*-TiO_2_(110) surfaces (red) that were saturated with EtOH at 290 K. Full lines are exponential fits to the data points, and the shaded areas display the standard deviations of these fits. Contributions of the non-reactive EtO_S_ ethoxides (*I*_*S*_) have been subtracted from the integrated intensity *I*_*m*_ of the measured C*1s* spectra. Accordingly, the plot shows the contributions to the C*1s* signals arising exclusively from the EtOH_Ti_ and EtO_Ti_ species, *I*_*Ti*_ (C*1s*).

To compare the photo-assisted dehydrogenation of EtOH_Ti_ and EtO_Ti_ species on the terraces of reduced and oxidized TiO_2_(110) surfaces in more depth, we collected PE C*1s* spectra at the ASTRID storage ring in Aarhus^54^. In the first series of PES experiments we started with an *o*-TiO_2_(110) surface, and in the second series with an *h*-TiO_2_(110) surface (Fig. 5). Starting with *h*-TiO_2_(110) was preferred over *r*-TiO_2_(110), because the acquisition of the PES data took long time and thus an *r*-TiO_2_(110) surface would gradually be changed into *h*-TiO_2_(110)^40^. Each series of experiments was conducted in UHV and repeated two times. The sample temperature was throughout kept at 290 K. The two C*1s* signals in the PE spectra arise from the carbon atoms in the –CH_2_O– and –CH_3_ groups^28^,^30^,^44^. As shown in Fig. 5a for *o*-TiO_2_(110), the C*1s* signals attenuated upon UV-light illumination. However, the C*1s* signal did not reach zero, even after UV-light illumination for 11 min. Because we know from our STM studies that the EtO_S_ ethoxides do not photo-react and no EtO_br_ species exist on EtOH/*o*-TiO_2_(110), we consider the C*1s* signal detected after 11 min illumination time to be exclusively due to the EtO_S_ ethoxide species. Before the illumination of the EtOH/*o*-TiO_2_(110) surface, the C*1s* signal originates from EtOH_Ti_ and EtO_Ti_ species on the terraces (*I*_*Ti*_), as well as EtO_S_ species bound along the step edges (*I*_*S*_).

Figure 5b shows the attenuation of the C*1s* signals for EtOH/*o*- and EtOH/*h*-TiO_2_(110) surfaces as function of the illumination time (filled red and green dots). To extract the contributions to the C*1s* signals arising solely from the EtOH_Ti_ and EtO_Ti_ species, *I*_*Ti*_ (C*1s*), we subtracted the signal arising from the EtO_S_ ethoxides, *I*_*S*_ (C*1s*) from the measured signal, *I*_*m*_(C*1s*). It can be seen that *I*_*Ti*_(C*1s*) attenuates exponentially on both TiO_2_(110) surfaces, with the attenuation on *o*-TiO_2_ being more than two times faster than on *h*-TiO_2_ (see the solid lines in Fig. 5b). Thus, also the PES data show that *o*-TiO_2_(110) surfaces are advantageous for the photo-assisted dehydrogenation of EtOH. Figure 5b further shows that the influence of the X-rays on the C*1s* signals (open dots) is measurable but negligibly small with the used low-intensity X-ray beam.

### DFT modelling of the EtOH photo-reaction on TiO_2_(110)

**Figure 6:**
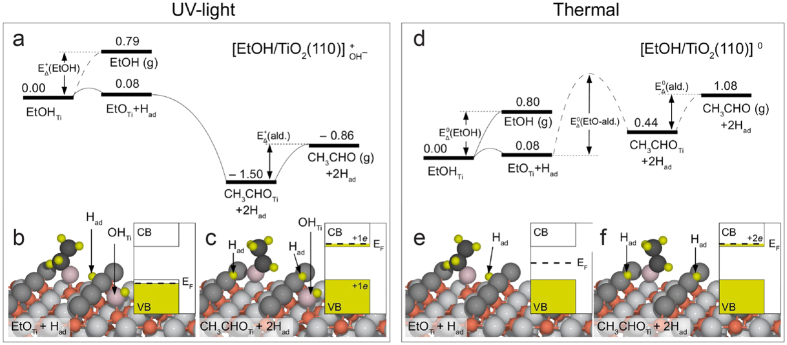
DFT modelling of the acetaldehyde formation on TiO_2_(110). Energy profiles for EtOH_Ti_ adsorbed on 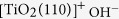 (**a**) and [TiO_2_(110)]^0^ (**d**) supercells. The formation of EtO_Ti_ ethoxides is considered in (**b**,**e**), and the formation of acetaldehyde species (CH_3_CHO_Ti_) is addressed in (**c**,**f**). All adsorption energies are given in eV. Small red-brown balls represent 5f-Ti atoms, large dark-grey balls O_br_ atoms and large light-grey balls in-plane O atoms. Atoms of the adsorbates are displayed as follows: C atoms: black balls; H_ad_ species: small yellow balls; O atoms: large pink balls. Corresponding band schemes, each consisting of the conduction (CB) and valence band (VB), are shown in the insets of (**b**,**c**,**e**,**f**). Filling of the bands is indicated by dark yellow colour, and positions of the Fermi level (E_F_) are indicated by dashed lines.

To further explore the photo-oxidation of EtOH on TiO_2_(110) in different oxidation states we performed first-principles DFT calculations (Fig. 6). In our theoretical analysis we assume that the photo-reaction of EtOH on TiO_2_ proceeds via oxidation by photo-generated holes and that the photo-electrons are trapped at the surface^2^,^3^,^4^. As possible trapping sites for electrons and holes we considered the splitting of a neutral water molecule in OH and H fragments that adsorb on 5f-Ti sites and O_br_ atoms, respectively. The two fragments act as trapping sites for electrons (OH_Ti_)^55^,^56^ and holes (H_ad_)^55^, respectively.

First, we studied the effect of the hole on the photo-reaction of EtOH (Fig. 6a–c). To this end, we used a stoichiometric TiO_2_(110) supercell with an OH_Ti_ group adsorbed on one side, denoted 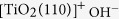. In order to maintain the hole created by the addition of the OH_Ti_ group, it is essential to use a stoichiometric rather than a reduced supercell^57^. In agreement with previous computational results^31^,^32^,^34^,^36^ and TPD data^27^,^28^,^29^,^39^ we found that the adsorption strength of molecularly (−0.79 eV) and dissociatively (−0.71 eV) adsorbed ethanol is strongest on-top on 5f-Ti sites and almost degenerated. Because the barrier for EtOH_Ti_ dissociation via O–H bond scission is only ~0.28 eV^32^ we consider the EtO_Ti_ ethoxide (Fig. 6b) as the given intermediate for acetaldehyde (CH_3_CHO_Ti_) formation (Fig. 6c). The reaction of the EtO_Ti_ ethoxide to CH_3_CHO_Ti_ is initiated by breaking of the C_1_–H bond, followed by H transfer to the O_br_ row and completed upon formation of the C_1_ = O double bond. On the 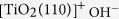 supercell (Fig. 6a), this reaction is barrier-less (i.e., it occurs spontaneously) and extremely favourable (−1.50 eV). The alternative pathway, the desorption of EtOH_Ti_ into the gas phase, is hindered by a barrier of ~0.79 eV [cf. 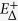 (EtOH) in Fig. 6a]. Thus, the photo-induced dehydrogenation of EtO_Ti_ ethoxide to CH_3_CHO_Ti_ is the preferred reaction on 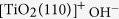. Once it has formed, a CH_3_CHO_Ti_ molecule cannot recombine again to EtO_Ti_ ethoxide, because this process is hindered by a ~1 eV high barrier. Apparently, this computational approach leads to results that are in good agreement with the experimental results on the EtOH photo-reaction.

For comparison, we also studied the interaction of EtOH_Ti_ and EtO_Ti_ species with a stoichiometric supercell without an OH_Ti_ group, which is denoted [TiO_2_(110)]^0^ (Fig. 6d–f). This was done to simulate the thermally activated dehydrogenation reaction as opposed to the photo-catalytic reaction. For this supercell we found that the reaction of EtO_Ti_ to CH_3_CHO_Ti_ is hindered by a high barrier, ~1.42 eV [cf. 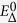 (EtO-ald.) in Fig. 6d], which is in good general agreement with experimental results for dehydrogenation reactions on metal oxides^7^. Accordingly, on [TiO_2_(110)]^0^, desorption of EtOH_Ti_ is more favourable than the formation of CH_3_CHO_Ti_ [cf. 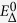 (EtOH)]. On both supercells considered, [TiO_2_(110)]^0^ and 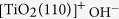, the CH_3_CHO_Ti_ molecules are rather weakly bound to 5f-Ti sites via the oxygen lone pairs (Fig. 6c,f). With both supercells we computed adsorption energies of −0.64 eV [cf. 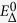 (ald.) and 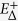 (ald.), respectively], and the ejection of CH_3_CHO_Ti_ into the gas phase is more favourable than the reconversion to the initial EtO_Ti_ state. Accordingly, acetaldehyde is expected to desorb at 290 K, the temperature at which we studied the photo-reaction of EtOH. Thus, these DFT calculations are in good agreement with the experimental observations.

To elucidate the reason why an EtO_Ti_ species on a 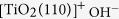 supercell reacts favourably to CH_3_CHO_Ti_ but not on [TiO_2_(110)]^0^ we studied the electronic structure of the considered EtO_Ti_ and acetaldehyde species, the results of which are shown in the insets of Fig. 6b,c,e,f. When an EtO_Ti_ reacts to CH_3_CHO_Ti_, the overall electronic effect is the transfer of two electrons from the EtO_Ti_ ethoxide to TiO_2_ states. Thus, for each photon two electrons are created – an effect that is well documented in the literature and often referred to as the “current doubling effect”^58^,^59^,^60^. On the 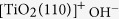 supercell (insets in Fig. 6b,c), one of the two electron is captured by the (photo-generated) hole, leading to electron transfer to the top of the valence band (VB), and the other is transferred to the TiO_2_ conduction band (CB). In contrast, on [TiO_2_(110)]^0^, both electrons are transferred to the TiO_2_-CB (insets in Fig. 6e,f), which costs more energy. That is, the reaction is favoured on 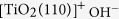 because energy is gained when one of the electrons is transferred to the top of the TiO_2_-VB rather than to the much higher level at the TiO_2_-CB.

## Discussion

The experimental results addressing the photo-reaction of EtOH on rutile TiO_2_(110) presented above are consistent with the following scheme: First, EtO_Ti_ species 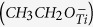 react with holes (h^+^), leading to the formation of ethoxide radicals:


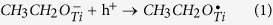


The ethoxide radicals react further via proton and electron transfer, leading to the formation of acetaldehyde and H_ad_ species:


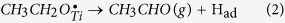


Such a direct oxidation of EtOH on TiO_2_(110) by the holes is fully supported by our DFT modelling. Further, this scheme is in line with previous spectroscopic studies, wherein EtOH and MeOH are reported to act as effective hole traps in photo-catalysed reactions^58^,^59^,^60^,^61^,^62^. Our results obtained on model surfaces in UHV are thus in remarkable agreement with TiO_2_ powder studies where TiO_2_ nanoparticles (anatase and rutile) were used to oxidize EtOH condensed as liquid or in the gas phase^13^,^14^,^15^,^16^,^17^,^18^,^19^,^20^,^21^. Most interestingly, two active sites have been proposed in the EtOH photo-reaction on the TiO_2_ particles, leading to the formation of acetaldehyde and formate/acetate, respectively^13^,^14^,^15^,^16^,^17^,^18^,^19^,^20^,^21^. These two active sites are distinct because of large differences in the observed reaction rates. This comparison suggests that the rutile TiO_2_(110) surface may serve as a good model for TiO_2_ nanoparticles that are used in many applications.

Another important result of our study is that the photo-reaction proceeds faster when the EtOH/EtO species are bound to regular surface Ti sites – the 5f-Ti sites. In fact, our STM studies uncover large differences in photo-reactivity between the various EtOH-related species on the TiO_2_(110) surface (Figs 1, 3 and 4). Whereas the EtOH_Ti_/EtO_Ti_ species were so highly reactive that even the low-intensity X-rays led to the removal of some adsorbates (Fig. 5b), the EtO_br_ species photo-reacted only in oxygen (Fig. 3) and the EtO_S_ species did not react at all at the tested conditions (Fig. 4). We note that such differences, although very clearly seen by STM, are very difficult to recognize in spectroscopic studies.

The observed low photo-reactivity of the ethoxides strongly bound at O_br_ vacancies and step edges may suggest that these adsorption sites constitute only a minor channel within the overall photo-reaction of EtOH on TiO_2_(110). Previous work^63^ on the photo-oxidation of trimethyl acetate [TMA or (CH_3_)_3_CCOO] on *r*-TiO_2_(110) showed an inhibition of hole-mediated photo-chemistry when the molecules are bound to O_br_ vacancies. These two examples [the TMA/*r*-TiO_2_(110)^63^ and EtOH/*r*-TiO_2_(110) – this work] indicate that the site requirement is independent of the specific organic molecule. We thus propose that regular surface Ti sites are mostly the active sites for hole-mediated photo-reactions on TiO_2_. Moreover, the step sites may contribute in the photo-reactivity in cases where the adsorption of the molecules is the limiting factor.

Among the studied TiO_2_(110) surfaces the EtOH_Ti_ and EtO_Ti_ species photo-reacted best on *o*-TiO_2_(110), in agreement with previous reports on other photo-catalysed reactions on rutile TiO_2_(110)^4^,^11^,^64^. From PES studies it is well-established that the adsorption of oxygen on reduced TiO_2_ surfaces leads to the depopulation of the Ti*3d* defect state in the band gap and an upward bending of the VB and CB’s^1^,^41^,^65^. This upward band bending near the TiO_2_(110) surface facilitates the charge carrier separation and drives the photo-generated holes towards the surface, thereby increasing the photo-reaction rate of hole-mediated reactions at the surface^65^. Furthermore, the depopulation of the defect state decreases the number of electrons in the near surface region – thus the rate for the recombination of holes and electrons is decreased. In addition, we note that the presence of O_ot_ adatoms on the TiO_2_(110) surface probably shifts the mix of EtOH_Ti_/EtO_Ti_ species more towards the EtO_Ti_ side, which should also facilitate the formation of acetaldehyde^17^.

Comparing the presented data addressing EtOH/TiO_2_(110) to those reported recently for MeOH/TiO_2_(110)^22^,^23^,^24^,^25^,^26^ we find both similarities and differences. Common in these studies and in our work is that the 5f-Ti sites are considered as important sites for photo-catalytic reactions on TiO_2_(110). In addition, methoxy groups are reported to be active species in photochemical hole scavenging reactions of MeOH on TiO_2_(110)^22^, similarly as we find that ethoxides in the Ti troughs are photo-active in case of EtOH/TiO_2_(110). However, Zhou *et al.* reported time-dependent 2PPE measurements, indicating that defects have a strong positive effect on the photocatalytic kinetic process^25^. Specifically, it was suggested that “surface defects and/or subsurface defects on the TiO_2_(110) surface enhance the photo-catalyzed MeOH dissociation rate by significantly lowering the photoreaction barrier”^25^. This conclusion appears to be opposite to what we found for EtOH/TiO_2_(110). Further studies are needed to explore the similarities and differences between these two systems.

## Conclusions

We have studied site-specific photo-reactions of EtOH species on TiO_2_(110) in different oxidation states and showed that the photo-reaction of EtOH on TiO_2_(110) proceeds most efficiently when the reactants are adsorbed on regular surface Ti sites on TiO_2_(110) surfaces with O_ot_ adatoms. On the contrary, strongly bound ethoxides, either adsorbed in O_br_ vacancies or at defects sites associated with the step edges, photo-react very slowly. When the photo-reaction was carried out in UHV at 290 K, acetaldehyde desorbed into the gas phase, whereas the second product, H_ad_ species, stayed on the surface. On the basis of the presented atomistic insights and literature data we propose that regular surface Ti sites are the most active sites in photo-reactions on TiO_2_ surfaces. In contrast, low-coordinated defects sites on the terraces and at the step edges are least active and the presence of oxygen is required to induce photo-reactivity of molecules at these sites. The rutile TiO_2_(110) surface, providing both regular Ti sites and defect sites, may serve as a good model for studying relevant processes on TiO_2_ nanoparticles, since loosely and strongly bound adsorbates can be studied simultaneously.

## Methods

### Experimental details

The STM and TPD experiments were carried out in an UHV chamber with a base pressure in the low 10^−11^ Torr range equipped with a homebuilt, variable-temperature Aarhus STM, a QMS, and standard facilities for sample preparation and characterization^49^. The Aarhus STM used in this study is capable of fast scanning at high resolution in a temperature range between 100 and 400 K^66^. Electrochemically etched tungsten tips were used in all the STM experiments. The differentially pumped QMS (Balzers) was connected to the main chamber via a closed cone with a small aperture (d ~ 3 mm) facing the sample at a distance of ~1 mm. This design ensures that only molecules released from the sample can reach the QMS. The temperature of the sample could be varied from 100 K using liquid nitrogen to 1200 K by heating the back side of the sample with a filament and electron bombardment. The sample temperature (measured using a K-type thermocouple) was controlled and recorded with a Eurotherm temperature controller that contains an automatic compensation of ambient temperature changes.

To obtain clean TiO_2_(110) surfaces, the crystals were Ar^+^ sputtered at 300 K and vacuum annealed at 880–950 K several times. A short flash to ~600 K was applied when the sample reached 300 K after vacuum annealing in order to free the sample from water and H_ad_ species that result from water adsorption during the cooling of the sample after the 20 min anneal^46^. The used TiO_2_(110) samples were characterized by O_br_ vacancy densities between 6.1 and 10.8% ML. All the STM images presented in this study were acquired in the constant current mode with a tunnelling voltage of ~+1.25 V and a tunnelling current of ~0.1 nA. Throughout, the sample was kept between 110 and 135 K during scanning.

The analysis of the STM data corresponding to Figs 1 and 3 rely on scanned areas of at least ~3000 nm^2^ for each given density and the given error bars represent the *standard error* 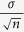, with 
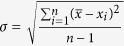
 being the *standard deviation* of the counted adsorbates, or O_br_ vacancies, and *n* being the number of the analysed STM images of the same size.

The PES C*1s* measurements were conducted at the ASTRID synchrotron radiation facility in Aarhus^54^ at the SGM1/Scienta beamline, using a low-intense synchrotron beam and a Scienta 200 analyser. The photon energy, *hν*, was 350 eV and the pass energy, *E*_*pass*_, was set to 75 eV. The PE C*1s* spectra were recorded at normal emission with the sample at 290 K. Great care was taken to avoid beam damage, which we identified to be a serious problem for this particular system and the chosen sample temperature. For each surface, *h*- and *o*-TiO_2_(110), respectively, three series of UV light experiments were carried out. Additionally, we conducted for each surface two series of control experiments to check for beam damage. The positions of the C*1s* peaks were calibrated using a gold reference. The C*1s* spectra were normalized to the photon flux and a linear background was subtracted. Subsequently, the spectra were fit with two Gaussian peaks to extract the integrated intensity *I*_*m*_ of the measured C*1s* spectra (the area of the Gaussian fits). For each surface, the three data series were fit with a global exponential function *I*(*t*) = *I*_*t*_(*t*)+*I*_*s*_, where *I*_*t*_(*t*) = *I*_*t,0*_ exp(−*t*/*τ*), and *I*_*t,0*_ and *I*_*s*_ were allowed to vary for each series, whereas the lifetime *τ* was constrained to be the same. The fits were weighted with the standard deviations of the C*1s* peak area.

For the EtOH exposures we used directional dosers, containing a 10 μm glass capillary array disk of ~8 mm diameter. For the exposures, the samples were placed in front of the doser at a distance of ~1 mm. When using such a microcapillary array doser, the local EtOH pressure at the TiO_2_(110) surface is unknown, but unwanted exchange reactions at the chamber walls are kept at a minimum.

The UV-light illumination was accomplished in all the experiments with an UV-A LED source (Optimax 365) with a peak emission at 365 nm (~3.4 eV) and a full-width-at-half-maximum (FWHM) of 10 nm. The LED source was mounted outside of the UHV chambers at a CF35 viewport, and the light intensity in front of the sample was estimated to be ~2 × 10^16^ photons/(s · cm^2^), which corresponds to ~20 mW/cm^2^.

### Computational details

The DFT calculations were performed using the GPAW program^67^,^68^, where the electrons are described using the projector augmented wave (PAW) method in the frozen core approximation^69^. The generalized gradient approximation (GGA) with the Perdew–Burke–Ernzerhof (PBE) functional^70^ was used to describe the exchange-correlation effects. The TiO_2_(110) surface was modelled using periodic slabs of four TiO_2_ tri-layers with a *c*(4 × 2) surface unit cell. All four tri-layers and the adsorbates were fully relaxed. The climbing nudged elastic band (NEB) method^71^ was used to calculate diffusion and dissociation barriers.

The presence of a photo-induced hole at the surface is modelled by introducing a co-adsorbed OH on one side of the slab. The OH is a spectator molecule and does not break or form any bonds during the reaction of EtOH. However, because of its strong electronegativity, the OH attracts an electron from the top of the VB on the stoichiometric slab, thereby producing a slab that is akin to that in the presence of a photo-induced hole^55^. This method has been used successfully in describing the photo-oxidation of methyl chloride over TiO_2_(110) surfaces^57^.

## Additional Information

**How to cite this article**: Hansen, J. Ø. *et al.* Unravelling Site-Specific Photo-Reactions of Ethanol on Rutile TiO_2_(110). *Sci. Rep.* **6**, 21990; doi: 10.1038/srep21990 (2016).

## Supplementary Material

Supplementary Information

## References

[b1] Diebold U. The surface science of titanium dioxide. Surf. Sci. Rep. 48, 53–229 (2003).

[b2] Thompson T. L. & Yates J. T. Jr. Surface science studies of the photoactivation of TiO_2_-new photochemical processes. Chem. Rev. 106, 4428–4453 (2006). 17031993 10.1021/cr050172k

[b3] Fujishima A., Zhang X. & Tryk D. A. TiO_2_ photocatalysis and related surface phenomena. Surf. Sci. Rep. 63, 515–582 (2008).

[b4] Henderson M. A. A surface science perspective on TiO_2_ photocatalysis. Surf. Sci. Rep. 66, 185–297 (2011).

[b5] Murdoch M. *et al.* The effect of gold loading and particle size on photocatalytic hydrogen production from ethanol over Au/TiO_2_ nanoparticles. Nat. Chem. 3, 489–492 (2011). 21602866 10.1038/nchem.1048

[b6] Ma Y. *et al.* Titanium dioxide-based nanomaterials for photocatalytic fuel generations. Chem. Rev. 114, 9987–10043 (2014). 25098384 10.1021/cr500008u

[b7] Idriss H. & Seebauer E. G. Reactions of ethanol over metal oxides. J. Mol. Catal. A 152, 201–212 (2000).

[b8] Rass-Hansen J., Falsig H., Jorgensen B. & Christensen C. H. Bioethanol: Fuel or feedstock? J. Chem. Technol. Biotechnol. 82, 329–333 (2007).

[b9] Surisetty V. R., Dalai A. K. & Kozinski J. Alcohols as alternative fuels: An overview. Appl. Catal. A Gen. 404, 1–11 (2011).

[b10] Dohnálek Z., Lyubinetsky I. & Rousseau R. Thermally-driven processes on rutile TiO_2_(110) – (1 × 1): A direct view at the atomic scale. Prog. Surf. Sci. 85, 161–205 (2010).

[b11] Henderson M. A. & Lyubinetsky I. Molecular-level insights into photocatalysis from scanning probe microscopy studies on TiO_2_(110). Chem. Rev. 113, 4428–4455 (2013). 23488875 10.1021/cr300315m

[b12] Pang C. L., Lindsay R. & Thornton G. Chemical reactions on rutile TiO_2_(110). Chem. Soc. Rev. 37, 2328–2353 (2008). 18818830 10.1039/b719085a

[b13] Sauer M. L. & Ollis D. F. Photocatalyzed oxidation of ethanol and acetaldehyde in humidified air. J. Catal. 158, 570–582 (1996).

[b14] Nimlos M. R., Wolfrum E. J., Brewer M. L., Fennell J. A. & Bintner G. Gas-phase heterogeneous photocatalytic oxidation of ethanol: Pathways and kinetic modeling. Environ. Sci. Technol. 30, 3102–3110 (1996).

[b15] Muggli D. S., McCue J. T. & Falconer J. L. Mechanism of the photocatalytic oxidation of ethanol on TiO_2_. J. Catal. 173, 470–483 (1998).

[b16] Muggli D. S., Lowery K. H. & Falconer J. L. Identification of adsorbed species during steady-state photocatalytic oxidation of ethanol on TiO_2_. J. Catal. 180, 111–122 (1998).

[b17] Hwang S. J. & Raftery D. *In situ* solid-state NMR studies of ethanol photocatalysis: Characterization of surface sites and their reactivities. Catal. Today 49, 353–361 (1999).

[b18] Wu W. C., Chuang C. C. & Lin J. L. Bonding geometry and reactivity of methoxy and ethoxy groups adsorbed on powdered TiO_2_. J. Phys. Chem. B 104, 8719–8724 (2000).

[b19] Liao L. F., Wu W. C., Chen C. Y. & Lin J. L. Photooxidation of formic acid vs formate and ethanol vs ethoxy on TiO_2_ and effect of adsorbed water on the rates of formate and formic acid photooxidation. J. Phys. Chem. B 105, 7678–7685 (2001).

[b20] Coronado J. M., Kataoka S., Tejedor-Tejedor I. & Anderson M. A. Dynamic phenomena during the photocatalytic oxidation of ethanol and acetone over nanocrystalline TiO_2_: simultaneous FTIR analysis of gas and surface species. J. Catal. 219, 219–230 (2003).

[b21] Yu Z. Q. & Chuang S. S. C. *In situ* IR study of adsorbed species and photogenerated electrons during photocatalytic oxidation of ethanol on TiO_2_. J. Catal. 246, 118–126 (2007).

[b22] Shen M. M. & Henderson M. A. Identification of the active species in photochemical hole scavenging reactions of methanol on TiO_2_. J. Phys. Chem. Lett. 2, 2707–2710 (2011).

[b23] Shen M. M. & Henderson M. A. Role of water in methanol photochemistry on rutile TiO_2_(110). J. Phys. Chem. C 116, 18788–18795 (2012).

[b24] Zhou C. Y. *et al.* Site-specific photocatalytic splitting of methanol on TiO_2_(110). Chem. Sci. 1, 575–580 (2010).

[b25] Zhou C. Y. *et al.* Effect of defects on photocatalytic dissociation of methanol on TiO_2_(110). Chem. Sci. 2, 1980–1983 (2011).

[b26] Guo Q. *et al.* Stepwise photocatalytic dissociation of methanol and water on TiO_2_(110). J. Am. Chem. Soc. 134, 13366–13373 (2012). 22794088 10.1021/ja304049x

[b27] Gamble L., Jung L. S. & Campbell C. T. Decomposition and protonation of surface ethoxys on TiO_2_(110). Surf. Sci. 348, 1–16 (1996).

[b28] Farfan-Arribas E. & Madix R. J. Role of defects in the adsorption of aliphatic alcohols on the TiO_2_(110) surface. J. Phys. Chem. B 106, 10680–10692 (2002).

[b29] Kim Y. K., Kay B. D., White J. M. & Dohnálek Z. Alcohol chemistry on rutile TiO_2_(110): the influence of alkyl substituents on reactivity and selectivity. J. Phys. Chem. C 111, 18236–18242 (2007).

[b30] Jayaweera P. M., Quah E. L. & Idriss H. Photoreaction of ethanol on TiO_2_(110) single-crystal surface. J. Phys. Chem. C 111, 1764–1769 (2007).

[b31] Nadeem A. M. *et al.* Ethanol photo-oxidation on a rutile TiO_2_(110) single crystal surface. Phys. Chem. Chem. Phys. 13, 7637–7643 (2011). 21225073 10.1039/c0cp01896a

[b32] Hansen J. Ø. *et al.* Direct evidence for ethanol dissociation on rutile TiO_2_(110). Phys. Rev. Lett. 107, 136102 (2011). 22026875 10.1103/PhysRevLett.107.136102

[b33] Martinez U. *et al.* Reduced step edges on rutile TiO_2_(110) as competing defects to oxygen vacancies on the terraces and reactive sites for ethanol dissociation. Phys. Rev. Lett. 109, 155501 (2012). 23102329 10.1103/PhysRevLett.109.155501

[b34] Muir J. N., Choi Y. & Idriss H. Computational study of ethanol adsorption and reaction over rutile TiO_2_(110) surfaces. Phys. Chem. Chem. Phys. 14, 11910–11919 (2012). 22832869 10.1039/c2cp40641a

[b35] Huo P. *et al.* Ethanol diffusion on rutile TiO_2_(110) mediated by H adatoms. J. Phys. Chem. Lett. 3, 283–288 (2012). 26285840 10.1021/jz201616z

[b36] Ma Z. B. *et al.* Photocatalytic dissociation of ethanol on TiO_2_(110) by near-band-gap excitation. J. Phys. Chem. C 117, 10336–10344 (2013).

[b37] Kershis M. D. & White M. G. Photooxidation of ethanol and 2-propanol on TiO_2_(110): evidence for methyl radical ejection. Phys. Chem. Chem. Phys. 15, 17976–17982 (2013). 24042847 10.1039/c3cp53027b

[b38] Kundu S. *et al.* Ethanol photoreaction on RuO_x_/Ru-modified TiO_2_(110). J. Phys. Chem. C 117, 11149–11158 (2013).

[b39] Walenta C. A. *et al.* Ethanol photocatalysis on rutile TiO_2_(110): the role of defects and water. Phys. Chem. Chem. Phys. 17, 22809–22814 (2015). 26264863 10.1039/c5cp03550c PMC4621531

[b40] Kurtz R. L., Stockbauer R., Madey T. E. & Román E. & de Segovia J. L. Synchrotron radiation studies of H_2_O adsorption on TiO_2_(110). Surf. Sci. 218, 178–200 (1989).

[b41] Wendt S. *et al.* The role of interstitial sites in the Ti3d defect state in the band gap of titania. Science 320, 1755–1759 (2008). 18535207 10.1126/science.1159846

[b42] Thomas A. G. *et al.* Comparison of the electronic structure of anatase and rutile TiO_2_ single-crystal surfaces using resonant photoemission and x-ray absorption spectroscopy. Phys. Rev. B 75, 035105 (2007).

[b43] Henderson M. A., Epling W. S, Peden C. H. F. & Perkins C. L. Insights into photoexcited electron scavenging processes on TiO_2_ obtained from studies of the reaction of O_2_ with OH groups adsorbed at electronic defects on TiO_2_(110). J. Phys. Chem. B 107, 534–545 (2003).

[b44] Kim Y. K. & Hwang C. C. Photoemission study on the adsorption of ethanol on clean and oxidized rutile TiO_2_(110)–(1 × 1) surfaces. Surf. Sci. 605, 2082–2086 (2011).

[b45] Hugenschmidt M. B., Gamble L. & Campbell C. T. The Interaction of H_2_O with a TiO_2_(110) Surface. Surf. Sci. 302, 329–340 (1994).

[b46] Wendt S. *et al.* Oxygen vacancies on TiO_2_(110) and their interaction with H_2_O and O_2_: A combined high-resolution STM and DFT study. Surf. Sci. 598, 226–245 (2005).

[b47] Wendt S. *et al.* Formation and splitting of paired hydroxyl groups on reduced TiO_2_(110). Phys. Rev. Lett. 96, 066107 (2006). 16606018 10.1103/PhysRevLett.96.066107

[b48] Kristoffersen H. H. *et al.* Role of steps in the dissociative adsorption of water on rutile TiO_2_(110). Phys. Rev. Lett. 110, 146101 (2013). 25167009 10.1103/PhysRevLett.110.146101

[b49] Lira E. *et al.* Dissociative and molecular oxygen chemisorption channels on reduced rutile TiO_2_(110): An STM and TPD study. Surf. Sci. 604, 1945–1960 (2010).

[b50] Lira E. *et al.* Effects of the crystal reduction state on the interaction of oxygen with rutile TiO_2_(110). Catal. Today 182, 25–38 (2012).

[b51] Matthiesen J. *et al.* Observation of all the intermediate steps of a chemical reaction on an oxide surface by scanning tunneling microscopy. ACS Nano 3, 517–526 (2009). 19309169 10.1021/nn8008245

[b52] Matthiesen J. *et al.* Formation and diffusion of water dimers on rutile TiO_2_(110). Phys. Rev. Lett. 102, 226101 (2009). 19658879 10.1103/PhysRevLett.102.226101

[b53] http://webbook.nist.gov/chemistry.

[b54] ISA, Centre for Storage Ring Facilities, Aarhus University, Aarhus: http://www.isa.au.dk.

[b55] Martinez U. & Hammer B. Adsorption properties versus oxidation states of rutile TiO_2_(110). J. Chem. Phys. 134, 194703 (2011). 21599078 10.1063/1.3589861

[b56] Deskins N. A., Rousseau R. & Dupuis M. Defining the role of excess electrons in the surface chemistry of TiO_2_. J. Phys. Chem. C 114, 5891–5897 (2010).

[b57] Kristoffersen H. H., Martinez U. & Hammer B. Modeling methyl chloride photo oxidation by oxygen species on TiO_2_(110). Top. Catal. 57, 171–176 (2014).

[b58] Gomes W. P., Freund T. & Morrison S. R. Chemical reactions involving holes at zinc oxide single crystal anode. J. Electrochem. Soc. 115, 818–823 (1968).

[b59] Micic O. I., Zhang Y. N., Cromack K. R., Trifunac A. D. & Thurnauer M. C. Photoinduced hole transfer from TiO_2_ to methanol molecules in aqueous-solution studied by electron-paramagnetic-resonance. J. Phys. Chem. 97, 13284–13288 (1993).

[b60] Yamakata A., Ishibashi T. & Onishi H. Electron- and hole-capture reactions on Pt/TiO_2_ photocatalyst exposed to methanol vapor studied with time-resolved infrared absorption spectroscopy. J. Phys. Chem. B 106, 9122–9125 (2002).

[b61] Ohno T., Izumi S., Fujihara K., Masaki Y. & Matsumura M. Vanishing of current-doubling effect in photooxidation of 2-propanol on TiO_2_ in solutions containing Fe(III) ions. J. Phys. Chem. B 104, 6801–6803 (2000).

[b62] Tamaki Y. *et al.* Direct observation of reactive trapped holes in TiO_2_ undergoing photocatalytic oxidation of adsorbed alcohols: Evaluation of the reaction rates and yields. J. Am. Chem. Soc. 128, 416–417 (2006). 16402821 10.1021/ja055866p

[b63] Wang Z. T., Deskins N. A., Henderson M. A. & Lyubinetsky I. Inhibitive influence of oxygen vacancies for photoactivity on TiO_2_(110). Phys. Rev. Lett. 109, 266103 (2012). 23368587 10.1103/PhysRevLett.109.266103

[b64] Cremer T., Jensen S. C. & Friend C. M. Enhanced photo-oxidation of formaldehyde on highly reduced o-TiO_2_(110). J. Phys. Chem. C 118, 29242–29251 (2014).

[b65] Zhang Z. & Yates J. T. Jr. Band bending in semiconductors: chemical and physical consequences at surfaces and interfaces. Chem. Rev. 112, 5520–5551 (2012). 22783915 10.1021/cr3000626

[b66] Lægsgaard E., Besenbacher F., Mortensen K. & Stensgaard I. A fully automated, thimble-size scanning tunnelling microscope. J. Microsc. 152, 663–669 (1988). 3254998 10.1111/j.1365-2818.1988.tb01435.x

[b67] Mortensen J. J., Hansen L. B. & Jacobsen K. W. Real-space grid implementation of the projector augmented wave method. Phys. Rev. B 71, 035109 (2005).

[b68] Enkovaara J. *et al.* Electronic structure calculations with GPAW: a real-space implementation of the projector augmented-wave method. J. Phys.-Condes. Matter. 22, 253202 (2010). 10.1088/0953-8984/22/25/253202 21393795

[b69] Blöchl P. E. Projector augmented-wave method. Phys. Rev. B 50, 17953–17979 (1994). 10.1103/physrevb.50.17953 9976227

[b70] Perdew J. P., Burke K. & Ernzerhof M. Generalized gradient approximation made simple. Phys. Rev. Lett. 77, 3865–3868 (1996). 10062328 10.1103/PhysRevLett.77.3865

[b71] Henkelman G., Uberuaga B. P. & Jónsson H. A climbing image nudged elastic band method for finding saddle points and minimum energy paths. J. Chem. Phys. 113, 9901–9904 (2000).

