# Supplementary material for: Unravelling Site-Specific Photo-Reactions of Ethanol on Rutile TiO2(110)
Source: Sci Rep. 2016 Feb 26;6:21990. doi: 10.1038/srep21990 (PMC4768110; doi:10.1038/srep21990)
Supplement: Supplementary Information [file srep21990-s1.pdf]

# Unravelling Site-Specific Photo-Reactions of Ethanol on Rutile $TiO_2$(110)

# Supporting Information

Jonas Ø. Hansen[†], Regine Bebensee[†], Umberto Martinez[†], Soeren Porsgaard[†],

Estephania Lira[†], Yinying Wei[†], Lutz Lammich[†], Zheshen Li[†], Hicham Idriss[‡],

Flemming Besenbacher[†], Bjørk Hammer[†], and Stefan Wendt[†,*]

[†]*Interdisciplinary Nanoscience Center (iNANO), Department of Physics and Astronomy, and Institute for Storage Ring Facilities (ISA), Aarhus University, DK-8000 Aarhus C, Denmark*

[‡]*SABIC-Centre for Research and Development (CRD) at KAUST, P.O. Box 4545-4700, Thuwal 23955, Saudi Arabia*

***Corresponding author:** swendt@phys.au.dk

1. Enlarged STM images of Fig. 1 in the main article: Figures S1 to S9.

2. Additional STM experiments addressing the assignments of surface species: Figure S10 and corresponding text.

3. Confirmation of $H_{ad}$ formation in the photo-reaction on EtOH / *r*-$TiO_2$(110) by TPD measurements: Figure S11 and corresponding text.

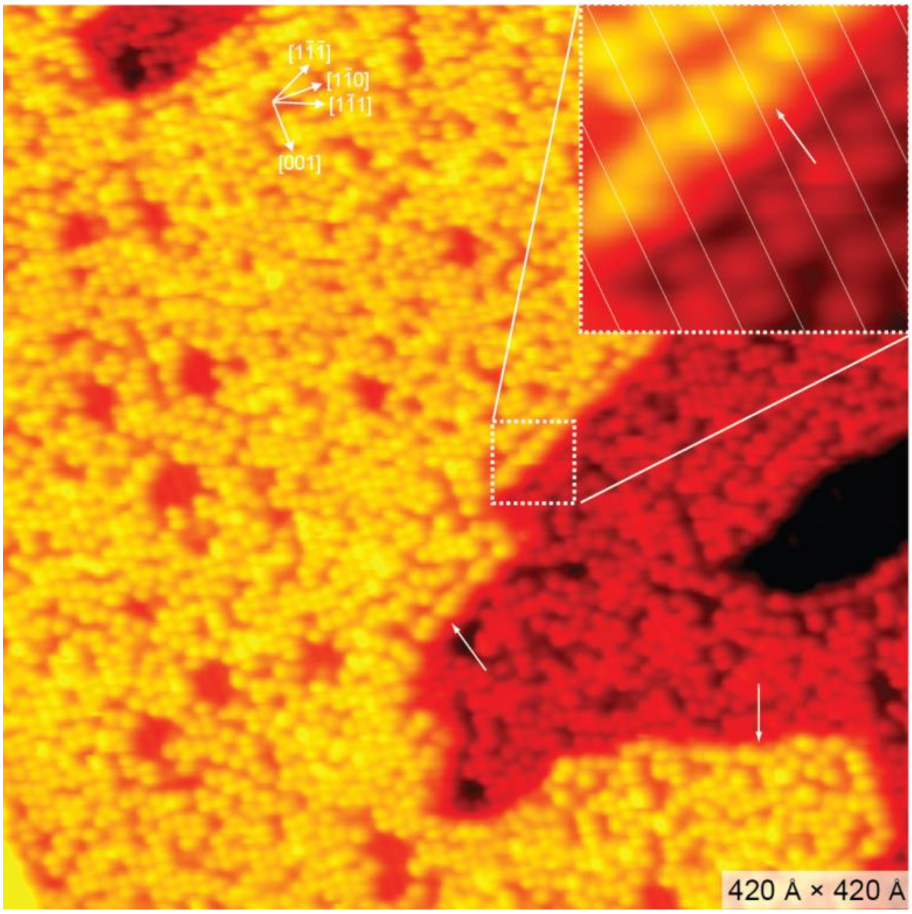


**Fig. S1 (see Fig. 1a):** STM image (420 Å × 420 Å) obtained after exposing an *r*-$TiO_2$(110) surface to EtOH at 300 K. Few of the $EtO_S$ ethoxides are indicated by white arrows. In the inset, the Ti troughs are indicated by thin white lines. The STM image was collected with a tunnelling current ≤ 0.1 nA and a tunnelling voltage of ~1.2 V.

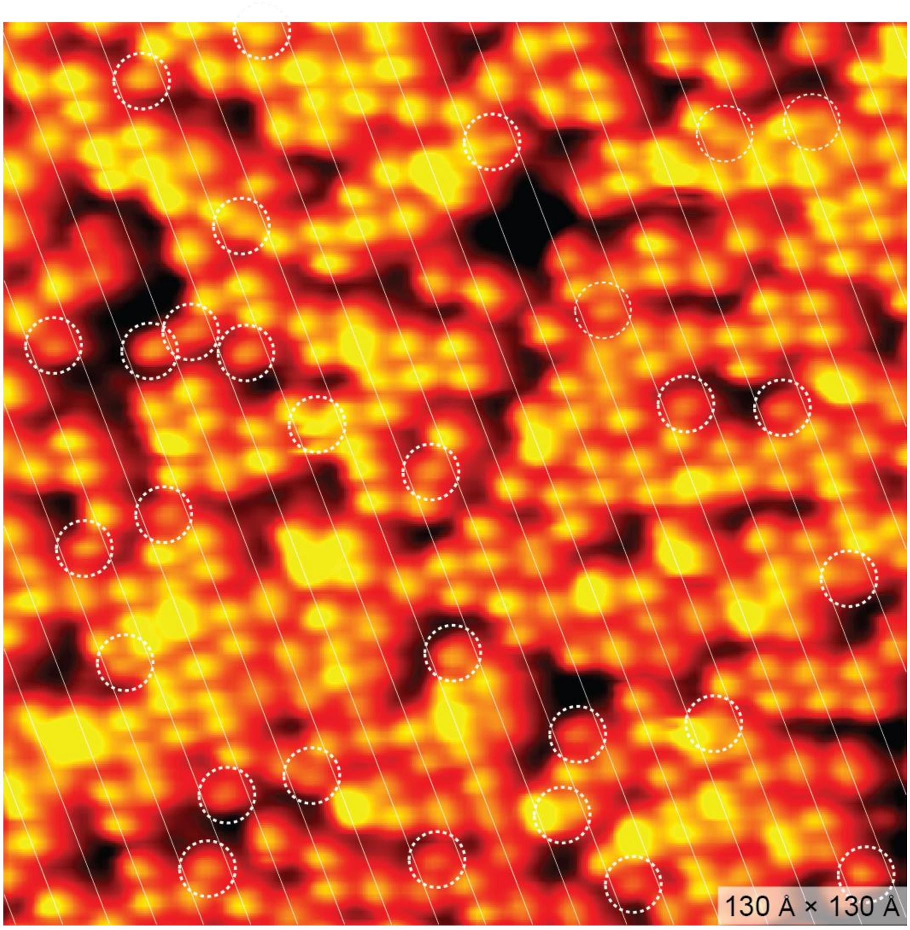


**Fig. S2 (see Fig. 1b):** STM image (130 Å × 130 Å) acquired within the same experiment as the image shown in Fig. S1 after exposing an $r$-$TiO_2(110)$ surface to EtOH at 300 K. The Ti troughs are indicated by thin white lines and $EtO_{br}$ ethoxides by white dotted circles. The STM image was collected with a tunnelling current ≤ 0.1 nA and a tunnelling voltage of ~1.2 V.

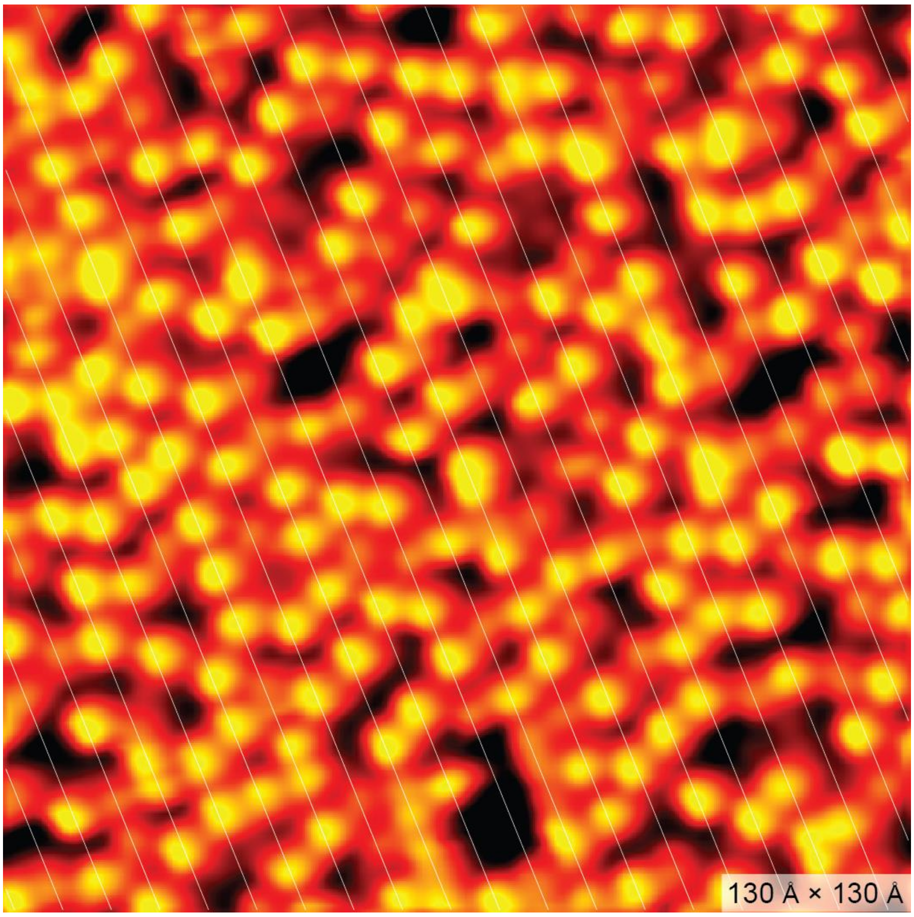


**Fig. S3 (see Fig. 1c):** STM image (130 Å × 130 Å) acquired after exposing an *o*-$TiO_2$(110) surface to EtOH at 300 K. The Ti troughs are indicated by thin white lines. Note that no $EtO_{br}$ ethoxides were formed on *o*-$TiO_2$(110), because no $O_{br}$ vacancies exist on this surface. The STM image was collected with a tunnelling current $\leq$ 0.1 nA and a tunnelling voltage of ~1.2 V.

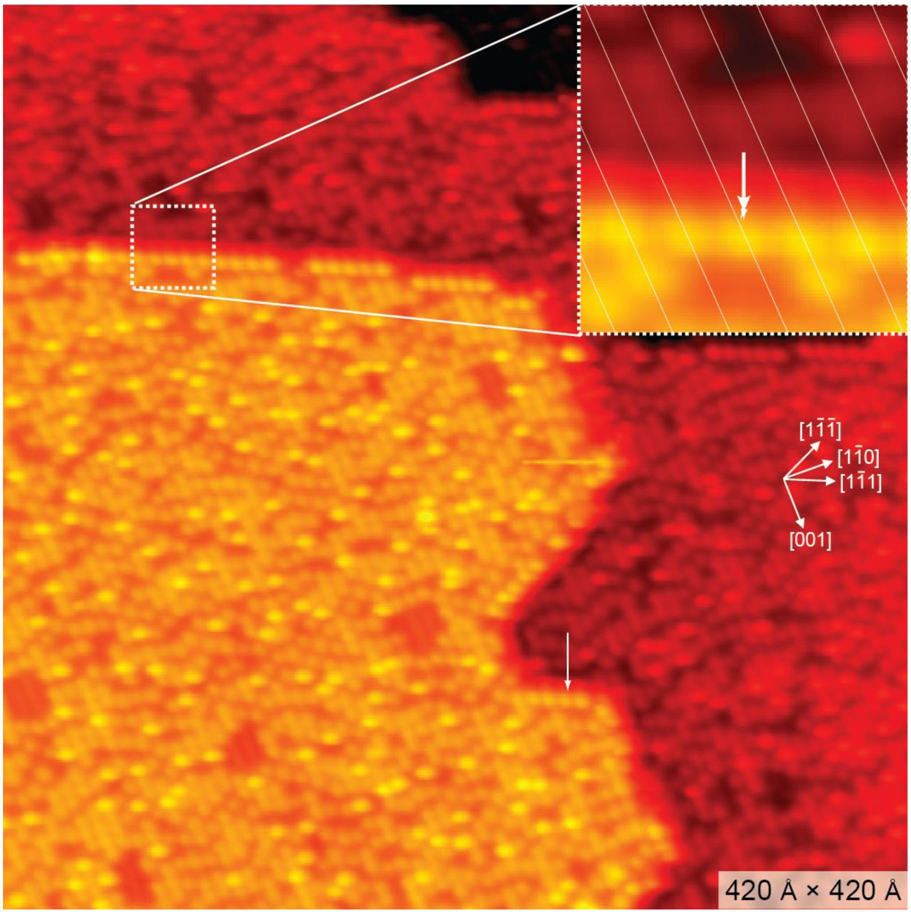


**Fig. S4 (see Fig. 1d):** STM image (420 Å × 420 Å) acquired after illumination of the EtOH-covered *r*-$TiO_2$(110) surface with UV-light for 11 min at 290 K in UHV. All the $EtOH_{Ti}$ / $EtO_{Ti}$ species disappeared but not the $EtO_{br}$ and $EtO_S$ ethoxides. Few of the $EtO_S$ ethoxides are indicated by white arrows. In the inset, the Ti troughs are indicated by thin white lines. The STM image was collected with a tunnelling current ≤ 0.1 nA and a tunnelling voltage of ~1.2 V.

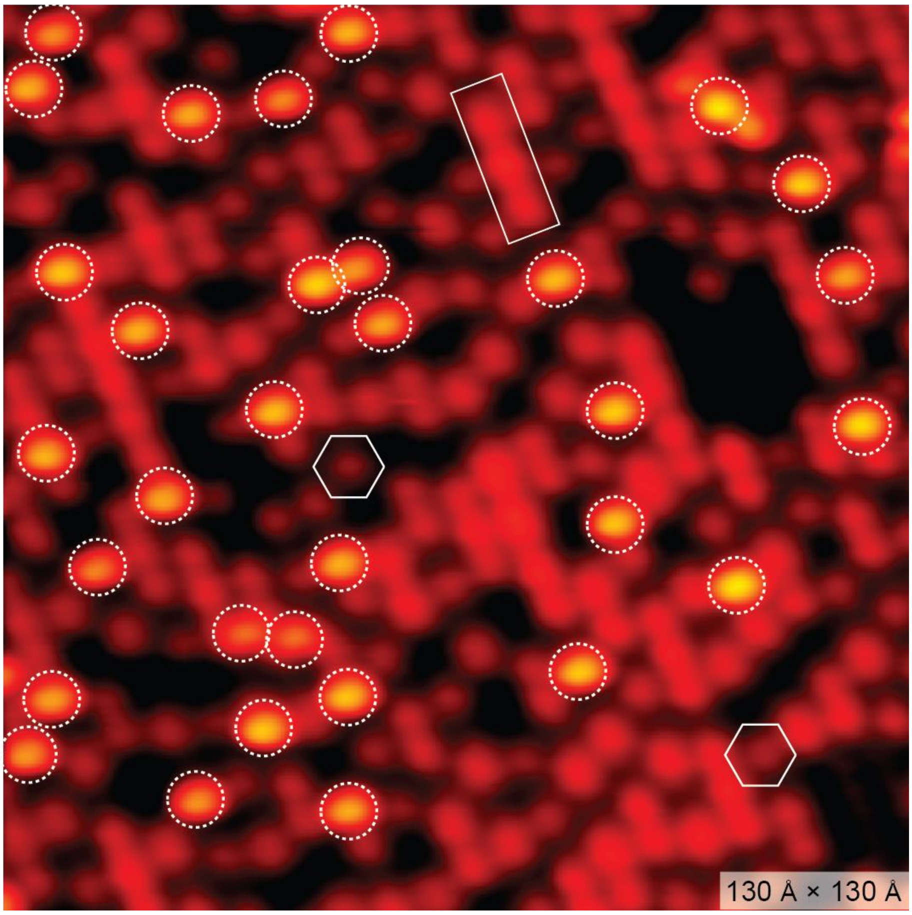


**Fig. S5 (see Fig. 1e):** STM image (130 Å × 130 Å) acquired within the same experiment as the image shown in Fig. S4 after exposing an *r*-$TiO_2(110)$ surface to EtOH at 300 K and UV light illumination. White dotted circles indicate $EtO_{br}$ ethoxides. Few isolated $H_{ad}$ species are indicated by hexagons and "rows of $H_{ad}$ species" by rectangles. Within the rows of $H_{ad}$ species each other $O_{br}$ is capped with an $H_{ad}$ species. The STM image was collected with a tunnelling current ≤ 0.1 nA and a tunnelling voltage of ~1.2 V.

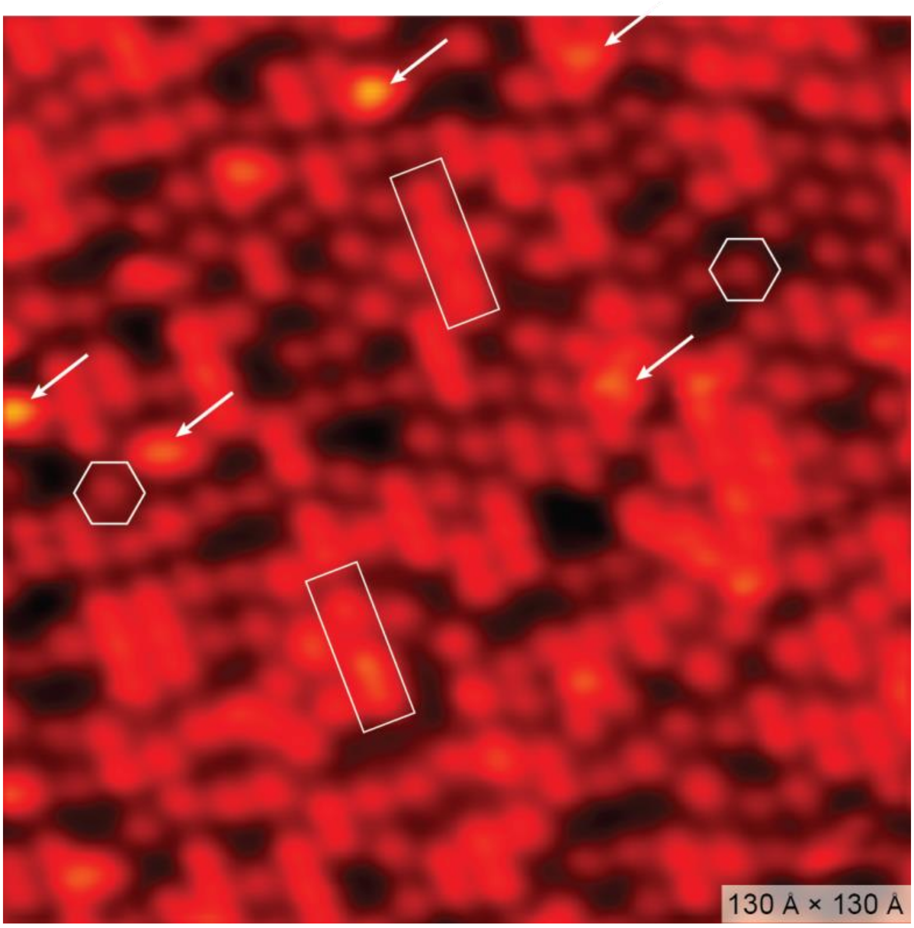


**Fig. S6 (see Fig. 1f):** STM image (130 Å × 130 Å) obtained after illumination of an EtOH-covered *o*-$TiO_2$(110) surface with UV-light for 11 min at 290 K in UHV. Few isolated $H_{ad}$ species are indicated by hexagons and "rows of $H_{ad}$ species" by rectangles. White arrows indicate water monomers. The STM image was collected with a tunnelling current ≤ 0.1 nA and a tunnelling voltage of ~1.2 V.

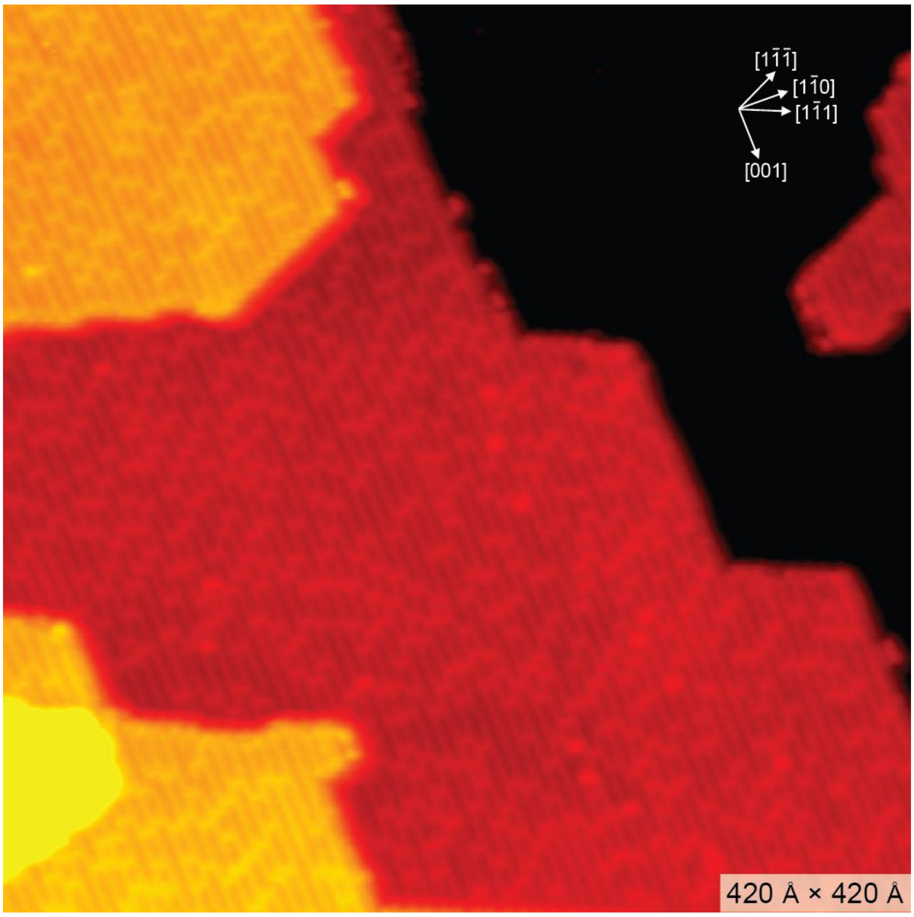


**Fig. S7 (see Fig. 1g):** STM image (420 Å × 420 Å) acquired after annealing of the $TiO_2(110)$ crystal for 2 min at ~550 K. The preceding preparations consisted of (i) saturation of *r*-$TiO_2(110)$ with EtOH at RT and (ii) UV-light illumination for 11 min at 290 K in UHV. A clean of *r*-$TiO_2(110)$ surface resulted characterized by an $O_{br}$ vacancy density about twice as high as the one found on the clean *r*-$TiO_2(110)$ surfaces at the very beginning of the experiment. The STM image was collected with a tunnelling current $\leq$ 0.1 nA and a tunnelling voltage of ~1.2 V.

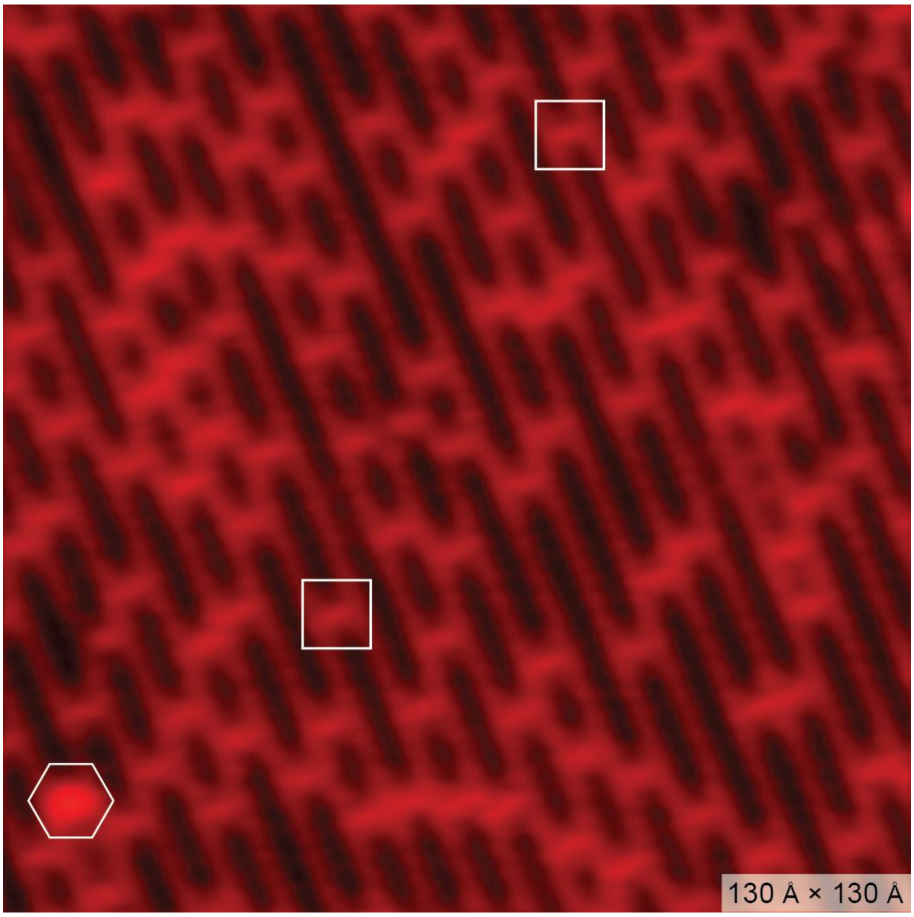


**Fig. S8 (see Fig. 1h):** STM image **(**130 Å × 130 Å) acquired after annealing of the $TiO_2(110)$ crystal for 2 min at ~550 K. The preceding preparations consisted of (i) saturation of *r*-$TiO_2(110)$ with EtOH at RT and (ii) UV-light illumination for 11 min at 290 K in UHV. A clean of *r*-$TiO_2(110)$ surface resulted characterized by an $O_{br}$ vacancy density about twice as high as the one found on the clean *r*-$TiO_2(110)$ surfaces at the very beginning of the experiment. Few $O_{br}$ vacancies are indicated by rectangles. Residual, isolated $H_{ad}$ species are indicated by hexagons. The STM image was collected with a tunnelling current ≤ 0.1 nA and a tunnelling voltage of ~1.2 V.

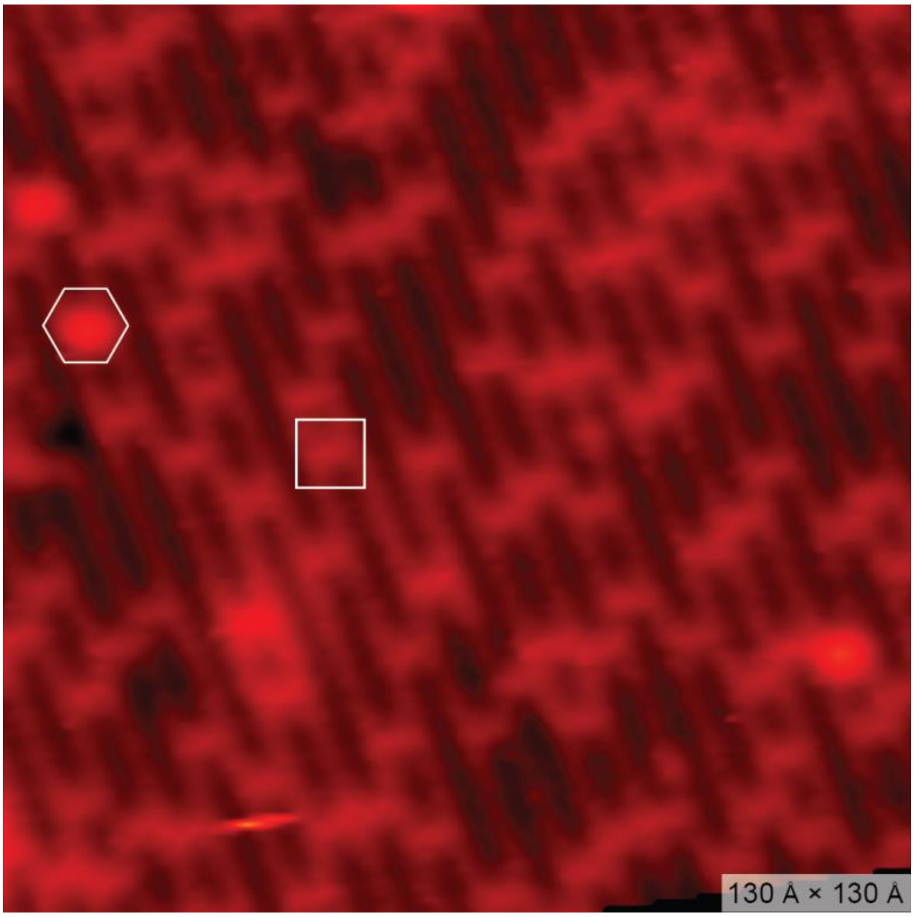


**Fig. S9 (see Fig. 1i):** STM image (130 Å × 130 Å) acquired after annealing of the $TiO_2(110)$ crystal for 2 min at ~630 K. The preceding preparations consisted of (i) saturation of *o*-$TiO_2(110)$ with EtOH at RT and (ii) UV-light illumination for 11 min at 290 K in UHV. A clean of *r*-$TiO_2(110)$ surface resulted characterized by an $O_{br}$ vacancy density about twice as high as the one found on the clean *r*-$TiO_2(110)$ surfaces at the very beginning of the experiment. Few $O_{br}$ vacancies are indicated by rectangles. Residual, isolated $H_{ad}$ species are indicated by hexagons. The STM image was collected with a tunnelling current ≤ 0.1 nA and a tunnelling voltage of ~1.2 V.

**Confirmation of the assignments in the STM experiments by means of scanning at high bias.** Considering the situation observed following UV-light illumination of EtOH / *r*-$TiO_2$(110), we performed several control experiments addressing the assignments of the observed surface species. For example, the assignment of the species in the $O_{br}$ rows to $H_{ad}$ species was further supported by the fact that they can be removed by $O_2$ exposure[1,2] and by scanning with a tunneling voltage of $V_t$ = +3V (see ref. 3). Regarding the latter type of control experiment, an example is shown in Fig. S10. First we scanned an area of 80 Å × 80 Å size with our usually used scanning conditions (tunnelling current $I_t \leq 0.1$ nA and a tunnelling voltage $V_t$ of ~1.2 V). The resulting STM image is shown in Fig. S10a. Subsequently, the same area was scanned again, but this time with $V_t$ = +3V. We then zoomed out to a larger scanning area (166 Å × 166 Å) and switched back to our usual scanning conditions (see Fig. S10b). In this image, the area that was scanned earlier with a high bias ($V_t$ = +3V) is indicated by a white broken square. It can be seen that the $EtO_{br}$ species were not removed during scanning with high bias. However, as expected[3], all the $H_{ad}$ species disappeared after scanning with $V_t$ = +3V. As a result, the $O_{br}$ rows and the Ti troughs became clearly visible in this area.

In the area scanned with high bias the white lines are superimposed on the Ti troughs. By extrapolating the white lines to the area outside the white broken square it becomes evident that the many new species that appeared after UV-light illumination ascribed to $H_{ad}$ species are indeed centered at the $O_{br}$ rows (i.e. between the lines), as expected for the $H_{ad}$ species[3]. Moreover, it can be seen that the $EtO_{br}$ are centered about the $O_{br}$ rows. Also this result is expected, since the $EtO_{br}$ species are formed via EtOH

dissociation at $O_{br}$ vacancy sites[4]. The described control experiment further supports the assignments given in the main text.

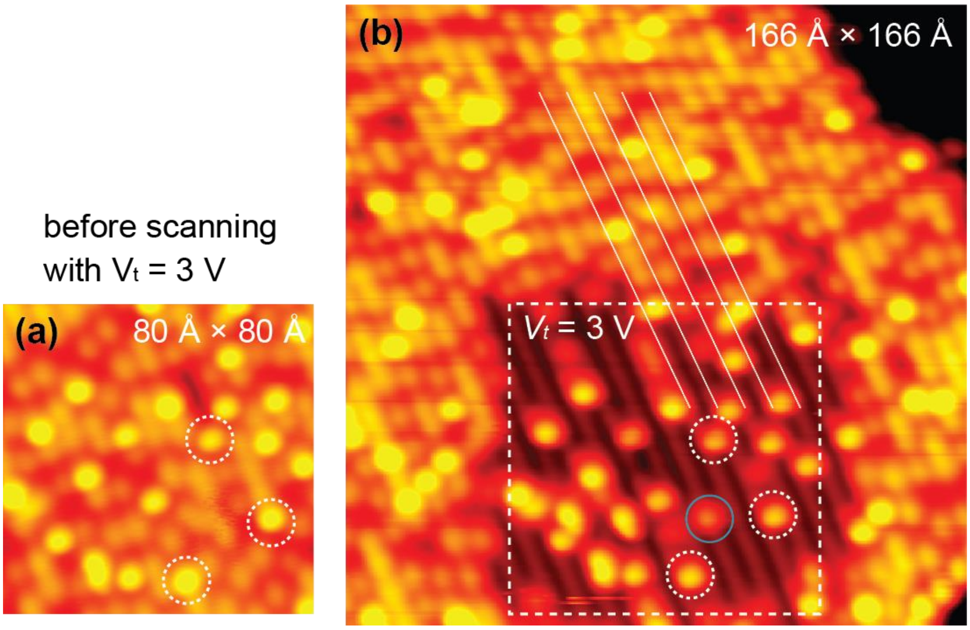


**Figure S10:** (a) STM image (80 Å × 80 Å) acquired within a similar experiment as the image shown in Fig. S4 after exposing an *r*-$TiO_2$(110) surface to EtOH at 300 K followed by UV light illumination at 290 K. The most apparent protrusions arise from $EtO_{br}$ ethoxides, three of which are indicated by white dotted circles. The other protrusions arise from $H_{ad}$ species. After this image was acquired, we scanned the same surface area again but this time with an increased bias of $V_t$ = +3 V, resulting in the removal of $H_{ad}$ species. (b) STM image (166 Å × 166 Å) acquired after scanning with $V_t$ = +3 V in the indicated area (same as in (a)). The white lines are superimposed on the Ti troughs in the squared region scanned with high bias. By extending these white lines to the area outside the square it becomes clear that the $EtO_{br}$ species and the $H_{ad}$ species are located at the $O_{br}$ rows between the Ti troughs (thin white lines), confirming their assignments. In (b), the same three $EtO_{br}$ species are indicated as in (a). In addition, a newly appeared species in a Ti trough (probably a water monomer from the background) is indicated by a blue circle.

**Confirmation of $H_{ad}$ formation by TPD measurements**. The water-TPD spectra (*m/z* = 18) displayed in Fig. S11 further corroborate the formation of $H_{ad}$ species in the photo-reaction on EtOH / *r*-$TiO_2$(110). The black TPD spectrum corresponds to EtOH / *r*-$TiO_2$(110) and was acquired after UV-light illumination for 11 min. This spectrum shows a small and narrow desorption feature at ~350 K (denoted "α") and a larger and broader feature with a maximum at ~520 K (denoted "β"). For comparison, we show a water-TPD spectrum acquired from an *h*-$TiO_2$(110) surface (green curve). This surface was prepared by exposing an *r*-$TiO_2$(110) surface to water, using the same $TiO_2$(110) sample. Here, the α-feature is similar as in the previous case, but the β-feature is less intense by a factor of ~2.6. The α-feature arises from water molecules in the residual gas, which adsorb in the Ti troughs[1,5] while cooling the sample down to ~100 K. The β-feature, however, arises from the recombination of $H_{ad}$ species with $O_{br}$ atoms[1,5]. Consequently, the integrated area of the β-feature is a measure of the $H_{ad}$ density.

An STM analysis of the *h*-$TiO_2$(110) surface corresponding to the green spectrum in Fig. S11 yielded an $H_{ad}$ density of (11 ± 0.5) %ML, which is a factor of ~2.45 less than on the illuminated EtOH / *r*-$TiO_2$(110) surface [(27 ± 1) %ML, see Fig. 1d,e]. Accordingly, the TPD and STM data are in excellent agreement.

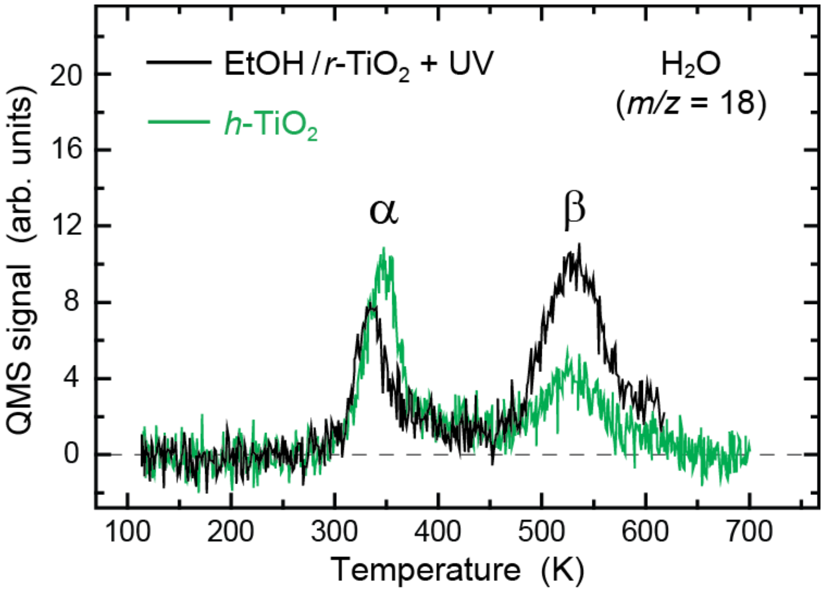


**Figure S11:** Water-TPD spectra of an illuminated EtOH / *r*-$TiO_2$(110) surface (black curve) and a non-illuminated *h*-$TiO_2$(110) surface (green curve). The same $TiO_2$(110) crystal was used for these experiments.

**References of the Supplemental Information:**


1. Henderson M. A., Epling W. S, Peden C. H. F. & Perkins C. L. Insights into photoexcited electron scavenging processes on $TiO_2$ obtained from studies of the reaction of $O_2$ with OH groups adsorbed at electronic defects on $TiO_2$(110). *J. Phys. Chem. B* **107**, 534–545 (2003).
2. Matthiesen J., *et al.* Observation of all the intermediate steps of a chemical reaction on an oxide surface by scanning tunneling microscopy. *ACS Nano* **3**, 517–526 (2009).
3. Wendt S., *et al.* Oxygen vacancies on $TiO_2$(110) and their interaction with $H_2O$ and $O_2$: A combined high-resolution STM and DFT study. *Surf. Sci.* **598**, 226–245 (2005).
4. Hansen J. Ø., *et al.* Direct evidence for ethanol dissociation on rutile $TiO_2$(110). *Phys. Rev. Lett.* **107**, 136102 (2011).
5. Hugenschmidt M. B., Gamble L. & Campbell C. T. The Interaction of $H_2O$ with a $TiO_2$(110) Surface. *Surf. Sci.* **302**, 329–340 (1994).
